# Wafer‐Scale Dendritic Nanoporous Platinum Films for Neural Electrodes

**DOI:** 10.1002/advs.78193

**Published:** 2026-10-06

**Authors:** Kyeongbin Kang, Joosung Oh, Soumen Dutta, Sun Woo Jang, Byeong Su Gu, Woosung Cho, Mingyu Kwak, Sunguk Hong, Seongjae Lee, Hyo Kyoung Jeon, Juwan Han, Keehoon Kim, Sung‐Min Park, Unyong Jeong, In Su Lee

**Affiliations:** ^1^ Center For Nanospace‐confined Chemical Reactions (NCCR) Pohang University of Science and Technology (POSTECH) Pohang South Korea; ^2^ Division of Advanced Materials Science Pohang University of Science and Technology (POSTECH) Pohang South Korea; ^3^ Department of Materials Science and Engineering Pohang University of Science and Technology (POSTECH) Pohang South Korea; ^4^ Department of Chemistry Pohang University of Science and Technology (POSTECH) Pohang South Korea; ^5^ Department of Mechanical Engineering Pohang University of Science and Technology (POSTECH) Pohang South Korea; ^6^ Major of Medical Science and Engineering Pohang University of Science and Technology (POSTECH) Pohang South Korea; ^7^ AX Graduate School Pohang University of Science and Technology (POSTECH) Pohang South Korea

**Keywords:** implantable bioelectrode, metallic nanofilm, resilient bio‐interface, two‐dimensional Pt, wafer‐scale synthesis

## Abstract

Two‐dimensional (2D) metallic nanosheets are promising for neural implants, but their application is hindered by limited lateral dimensions, poor mechanical compliance, and insufficient electrochemical stability. Here, we report a wafer‐scale wet‐chemical synthesis of an ultrathin, intrinsically porous dendritic platinum (Pt) film (*den‐2DPt_waf_*) that can be transferred onto flexible substrates for robust neural interfacing. Confined Pt growth on a layered double hydroxide (LDH)‐modified Si wafer produces a wafer‐sized, 6‐nm‐thick film that is vertically conductive but laterally insulating. As a bioelectrode (area = 4 × 10^−4^ cm^2^), *den‐2DPt_waf_* exhibits low impedance (∼45 Ω at 1 kHz), high charge storage capacity (∼12 mC cm^−2^), and high charge injection capacity (1.13 mC cm^−2^). It also maintains its performance after >10,000 bending cycles at a 2‐mm radius and under accelerated oxidative conditions (3% H_2_O_2_), outperforming conventional flat Pt films. In vivo, *den‐2DPt_waf_* electrodes enable effective vagus nerve stimulation, inducing immediate, current‐dependent reductions in rat breathing rate while maintaining stable activation thresholds and electrochemical and structural integrity over two weeks. This scalable porous Pt film provides a mechanically compliant and electrochemically resilient platform for long‐term neural recording and stimulation.

## Graphical abstract

Wafer‐scale dendritic nanoporous platinum films with intrinsic nanoscale porosity are synthesized by suraface‐confined wet chemistry and transferred onto flexible substrates to create tissue‐conformal neural electrodes. The ultrathin films combine mechanical flexibility with electrochemical stability, enabling low‐impedance, high‐capacitance interfaces for neural stimulation and long‐term bioelectronic integration.

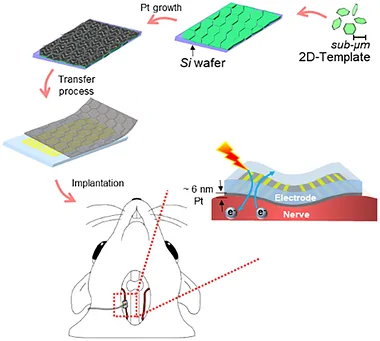

## 1 Introduction

Precise nanoscale engineering of two‐dimensional (2D) nanostructures has enabled abundant active‐site exposure, efficient charge transport, and exceptional interfacial adhesiveness, making them attractive for advanced catalytic platforms, energy systems, and implantable or wearable technologies [1, 2, 3, 4]. However, the range of materials that can be shaped into such ultrathin architectures remains limited [3, 5]. Among unconventional 2D nanostructures, platinum‐group metals have gained considerable attention for their outstanding electrocatalytic properties [6, 7]; yet, their potential in bioelectronic devices remains largely unexplored. This gap is striking given the long‐standing clinical use of bulk platinum (Pt), valued for their reliable performance in biological environments [8, 9], and the intrinsic mechanical compliance of 2D morphologies, which could promote integration on soft biological tissues.

Implantable bioelectrodes require interfacing materials that combine high conductivity, biocompatibility, and long‐term mechanical and electrochemical stability [10, 11, 12]. In particular, for electrodes intended for chronic electrical stimulation, ensuring electrochemical robustness during high charge injection remains a critical challenge because exceeding the electrochemical window can trigger irreversible processes such as water electrolysis and electrode degradation, compromising both device integrity and tissue safety; therefore, prioritizing electrode materials with high capacitance and low impedance is crucial [13, 14, 15, 16]. Common neural interface materials include platinum and various other metals [17, 18, 19], titanium nitride (TiN) [20], iridium oxide (IrO_x_) [21], and conducting polymers such as poly(3,4‐ethylenedioxythiophene):poly(styrenesulfonate) (PEDOT:PSS) [22]. Pt and its alloys are widely adopted in electrical stimulation and neuroprosthetics owing to their biostability and corrosion resistance in physiological environments; however, conventional evaporated Pt electrodes possess relatively smooth surfaces that provide limited effective interfacial area and low electrochemical capacitance, thereby restricting safe charge injection during neural stimulation [13, 23]. In contrast, IrO_x_ exhibits excellent charge injection capability for promising neural stimulation applications [24], whereas PEDOT:PSS can substantially reduce electrode impedance and improve electrode–tissue coupling [22]. Accordingly, planar Pt electrodes are often modified with conductive polymer coatings (e.g., PEDOT:PSS), Pt black, IrO_x_, or other nanostructured surface architectures to increase ion‐accessible surface area and charge storage capacity. Despite these advances, maintaining long‐term structural integrity, interfacial adhesion, and electrochemical stability remains an important consideration for flexible and chronically implanted devices, as polymer coatings may undergo swelling or delamination under physiological conditions [25, 26], whereas nanostructured metal coatings can be susceptible to mechanical damage, structural discontinuities, and localized electrochemical degradation during prolonged stimulation [27, 28, 29, 30].

In this context, nanostructured conductive interfaces have shown advantages for neural applications by increasing effective interfacial area and improving electrode–tissue coupling [31, 32, 33]. Accordingly, extending such nanoscale structural engineering to Pt represents a promising strategy for combining enhanced electrochemical performance with the intrinsic biostability of Pt. In addition, their multi‐scale conformal contact provides a structural basis for efficient neural stimulation without reliance on an overcoating layer [34]. Despite this promise, thin Pt nanostructures face fundamental barriers to scalable‐size adoption because achieving their anisotropic lateral growth is intrinsically challenging. Employing facet‐selective ligands [35], surfactant micelles [35, 36], or template confinement [37] has been investigated for growing 2D metallic structures, however lateral dimensions typically remain limited to the sub‐micron scale [38]. Furthermore, binder‐free 2D growth allowing device‐scale integration has not been achieved.

In this study, to achieve binder‐free synthesis of 2D Pt structures on wafer scale, we developed a wet chemical synthesis of Pt grown on a film of layered‐double‐hydroxide (LDH) nanoplates. The resulting Pt forms a continuous wafer‐scale film composed of laterally interconnected dendritic 2D Pt nanosheets, featuring an intrinsic nanoporous architecture with ∼3 nm gaps between adjacent dendritic branches. The Pt film was cleanly transferred onto flexible polymer substrates without structural damage. As a bioelectrode, the flexible nanosheet architecture is expected to enable conformal interfacing with neural tissue and improved electrode–tissue contact. The dendritic porosity enlarged the effective electrode^−^electrolyte interface, securing high charge storage capacity (CSC = 11.97 mC cm^−2^) and charge injection capacity (CIC = 1.13 mC cm^−2^), thereby enabling greater charge delivery within the operating potential range in physiological electrolytes and efficient charge transport. We demonstrated in vivo vagus nerve stimulation (VNS) in rats, stable stimulation current thresholds were maintained over a 2‐week implantation period while operating safely below the electrode's charge injection capacity limit.

## 2 Results and Discussion

### 2.1 Concept of Producing Wafer‐Scale Dendritic Two‐Dimensional Pt (*den‐2DPt_waf_*) and Its Use as Neuroelectrode

Figure 1 schematically illustrates the concept of producing wafer‐scale dendritic two‐dimensional Pt (*den‐2DPt_waf_*) and its integration as a flexible neuroelectrode for vagus nerve stimulation. Building on the previously reported LDH‐confined Pt synthesis, where dendritic Pt domains nucleate and grow on colloidal LDH nanoplates (lateral size ≈ 200 nm), the process was here extended to wafer‐scale by uniformly depositing the LDH plates over a SiO_x_/Si substrate to serve as a quasi‐2D growth template [39]. The resulting LDH layer exhibited a nearly flat topography with a root‐mean‐square roughness below 2 nm, which effectively suppressed step‐edge barriers during Pt domain growth and enabled the formation of a seamlessly interconnected, centimeter‐scale *den‐2DPt_waf_* film with an overall thickness of ∼6 nm and lateral coverage up to 5 × 5 cm^2^. The large‐area *den‐2DPt_waf_* film was then transferred onto flexible substrates via a poly(methyl methacrylate) [PMMA]‐assisted wet transfer, a widely adopted strategy for wafer‐scale 2D films. After spin‐coating a PMMA support layer, the LDH template was selectively removed by etching in a dilute hydrofluoric acid (HF) aqueous solution, allowing the liberated PMMA/*den‐2DPt_waf_* stack to float at the liquid surface. Separately, a polyimide (PI) (2 µm)/gold (Au) interconnect/PI (25 µm) was prepared and PI was dry‐etched through a mask to expose a target area of electrode array (0.04 mm^2^) to the air. The floating PMMA/*den‐2DPt_waf_* film was transferred onto the PI/Au interconnect/PI to electrically connect the *den‐2DPt_waf_* film to the underlying Au pattern, and then PMMA was dissolved. The Au interconnects were routed to an external stimulation device. Owing to the ultrathin Pt layer and micrometer‐scale total thickness (< ∼30 µm), the device behaved as a highly flexible and mechanically robust cuff electrode that could withstand severe bending without cracking or delamination. The resulting cuff‐type neuroelectrode was surgically implanted to gently wrap around the cervical vagus nerve, providing intimate conformal contact suitable for efficient electrical stimulation and long‐term bioelectronic interfacing.

**FIGURE 1:**
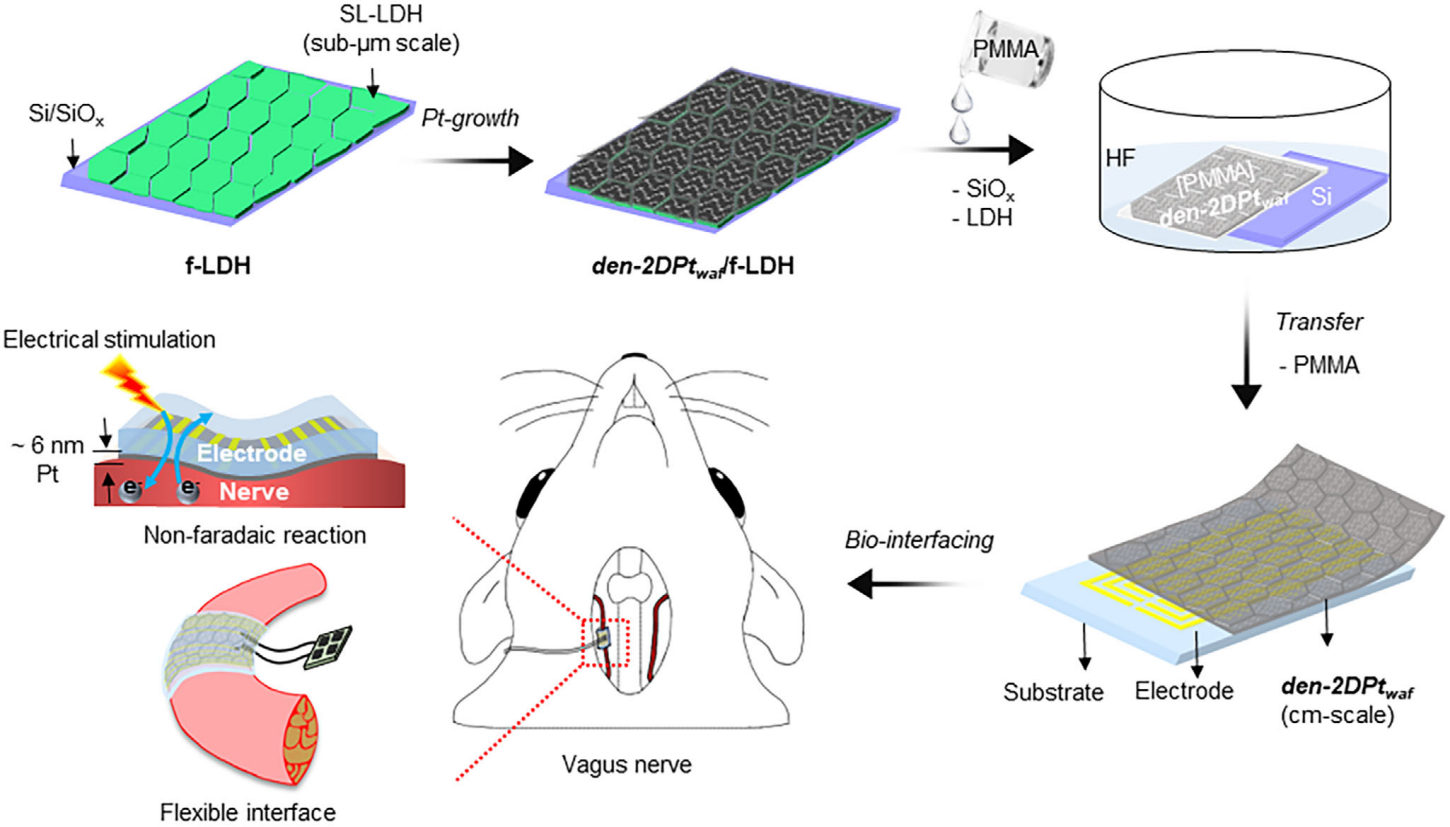
Wafer‐scale fabrication and transfer of dendritic 2D‐Pt (*den‐2DPtwaf*) for soft neural interfaces. Schematic illustration of *den‐2DPt_waf_* synthesis on SiOx/Si and transfer onto a PI (2 µm)/Au‐patterned/PI electrode. The resulting soft, implantable *den‐2DPt_waf_*/Au electrode enables conformal neural interfacing for vagus nerve stimulation.

### 2.2 Fabrication of Wafer‐Scale Dendritic Two‐Dimensional Pt (*den‐2DPt_waf_*) Layer

Figure 2a exhibits the procedure of growing the *den‐2DPt_waf_* film on the LDH template film. The surface‐confined growth of Pt was enabled by the metal‐ion–coordinating hydroxyl groups on the LDH sheets which would serve as a 2D template for nucleation and growth [39]. A centimeter‐scale LDH film (f‐LDH) was prepared by spin‐coating freshly synthesized ethanol dispersions of single‐layer NiCo‐LDH (SL‐LDH; lateral size = 260 ± 30 nm, thickness(*t*) ≈ 1 nm) with concentrations ranging from 5 ∼ 20 mg mL^−^^1^ (0.01 mL) onto a silicon oxide (SiO_x_)‐buffered Si(100) wafer (hereafter denoted as *Si*, 1 × 1 cm^2^) (Figure S1) [40]. The resulting LDH coatings were examined by atomic force microscopy (AFM) and scanning electron microscopy (SEM) to determine the optimum deposition conditions that yield a uniform and fully‐covered f‐LDH film on *Si* (Figure 2b (i, ii) and Figure S2), while suppressing random agglomeration or isolated island‐like clustering. AFM analysis (Figure 2b (iii)) revealed that the optimized f‐LDH/*Si* film, prepared using an SL‐LDH concentration of 20 mg mL^−^^1^, exhibited a low root‐mean‐square surface roughness (R_q_ = 1.2 nm over 25.0 µm^2^), indicating the formation of a smooth and continuous LDH coating with an average thickness of approximately 8.0 nm on *Si*. Subsequently, surface‐confined Pt growth was conducted by immersing the f‐LDH/*Si* film in an aqueous solution containing Na_2_PtCl_4_ and polyvinylpyrrolidone (PVP), followed by the addition of ascorbic acid (AA) and incubation at 70°C for 30 min (see Section 4.2 for experimental details). Pt layer continued to grow laterally on the LDH surface and eventually produced a continuous wafer‐scale *den‐2DPt_waf_* film covering the entire f‐LDH/*Si* surface. It was composed of densely tiled, hexagonal disc‐like den‐2DPt units that replicate the morphology and dimensions of the underlying LDH plates (Figure 2c (i, ii)). AFM revealed an increased step height for the as grown film (*t* ≈ 13 nm) compared with the underlying f‐LDH (*t* ≈ 8 nm), consistent with the formation of a crack free *den‐2DPt_waf_* overlayer with an average thickness of ∼5 nm and without uncontrolled three‐dimensional Pt agglomeration (Figure 2c (iii)). Cross‐sectional transmission electron microscopy (TEM) of focused ion beam (FIB)‐lamellae prepared from the multilayer *den‐2DPt_waf_*/f‐LDH/*Si* stack (Figure 2c (iv, v)), together with elemental line scan profiling, further confirmed a laterally uniform, continuous Pt rich layer with a thickness of ∼5.0 nm conformally coating the corrugated f‐LDH surface, corroborating the AFM derived thickness and other structural analyses.

**FIGURE 2:**
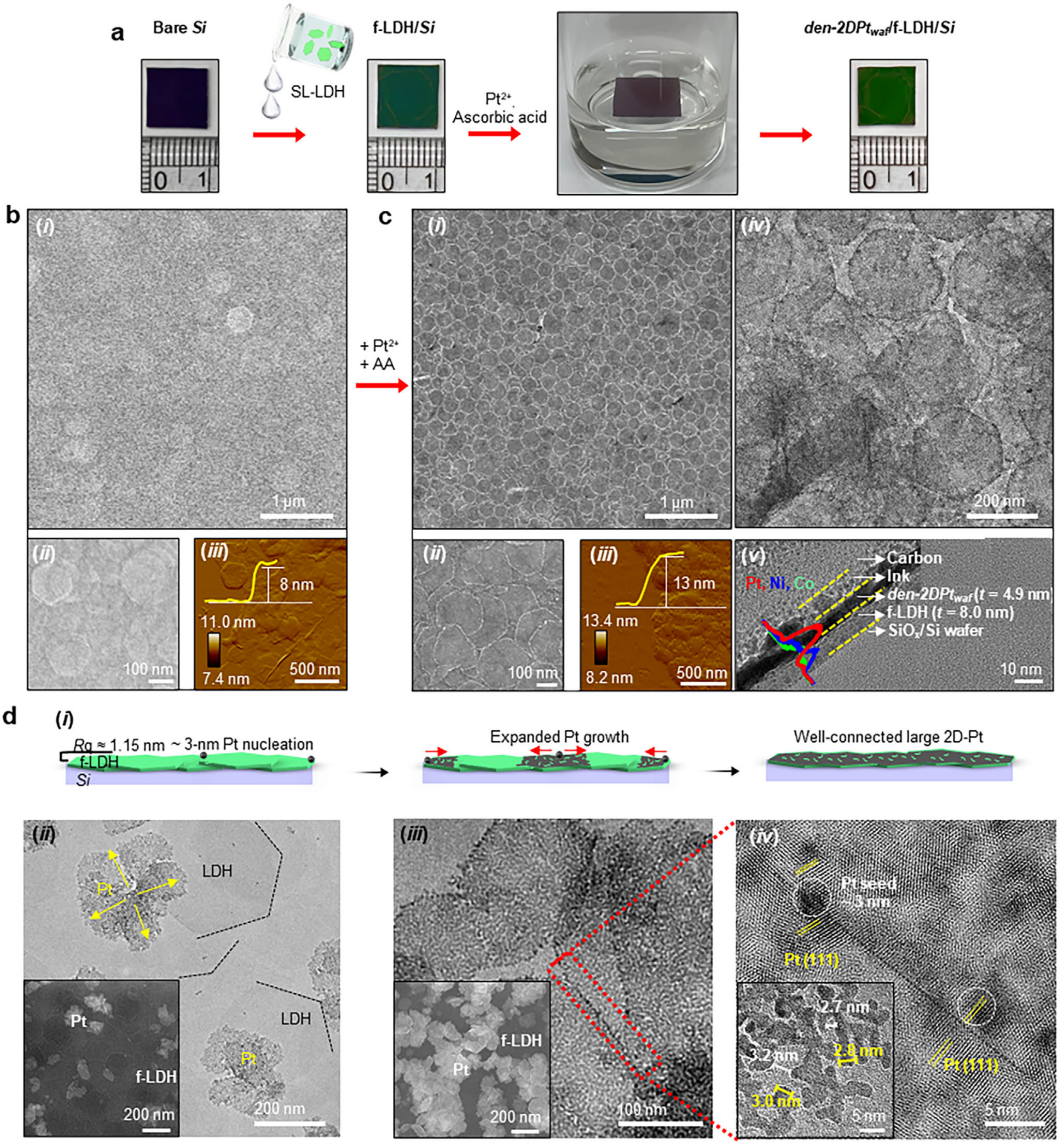
Formation and growth mechanism of the *den‐2DPtwaf* film enabled by a smooth LDH template. (a) Stepwise wet‐chemical synthesis of *den‐2DPt_waf_* on a spin‐coated single‐layer SL‐LDH film on SiO_x_/Si (i.e., *f‐LDH/Si*), yielding a continuous wafer‐scale Pt film. (b) SEM images at different magnifications (*i, ii*) and AFM height profile (*iii*) showing the smooth, low‐roughness *f‐LDH/Si* template. (c) SEM images (i, ii), AFM height profile (*iii*), TEM image (*iv*), and cross‐sectional TEM image with elemental mapping (*v*) of the resulting *den‐2DPtwaf/f‐LDH/Si*. (d) Schematic illustration of the proposed growth mechanism, showing that the low surface roughness of *f‐LDH/Si* enables barrier‐free Pt propagation across adjacent LDH domains (*i*), followed by Pt nucleation at LDH edges (*ii*), interconnection of the growing Pt sheets (*iii*), and their evolution into the characteristic branched and porous Pt architecture (*iv*).

The smooth surface provided uninterrupted cliff‐free surfaces for the growth of the *den*‐*2DPt*, facilitating seamless transition onto neighboring LDH plates while preserving the connectivity of 3 nm‐wide Pt branches (Figure 2d (i)). Time‐resolved SEM image revealed the emergence of sparsely distributed, nanoscale den‐2DPt patches (tens of nanometers in lateral size), which gradually expanded over the LDH plates (Figure 2d (ii); inset). TEM and high‐resolution TEM (HR‐TEM) of the Pt/LDH flakes detached from the *Si* substrate revealed that den‐2DPt growth, consisting of laterally interconnected Pt branches (∼3 nm in width), was initiated at the edges of the LDH plates (Figure 2d (iii, iv) and Figures S3 and S4). Since the edge and corner regions of LDH sheets generally exhibit higher chemical reactivity than the basal planes, Pt nucleation likely initiates preferentially at these sites [39, 41], from which growth proceeds inward along the smooth basal plane, with surface‐bound Pt(II) species directing 2D Pt extension across adjacent LDH domains. As the Pt fronts advanced across neighboring LDH plates, the initially discrete den‐2DPt domains merged into a single *den‐2DPt_waf_* film characterized with abundant in‐plane nanopores between dendritic branches (average gap ≈ 2.8 nm) and a highly uniform thickness over the entire 1 × 1 cm^2^ wafer area. Notably, the well‐recognized AA‐induced slow and continuous nucleation to yield ∼3‐nm Pt seeds, followed by rapid autocatalytic growth into uniform metallic Pt nanostructures, is also valid here, as observed in Figure 2d (iv) [36, 42]. Hence, the successful fabrication of large‐area 2D Pt, exceeding the current lateral‐size limitations, can primarily be attributed to the remarkably flat surface of f‐LDH/*Si* (R_q_ = 1.15 nm). Control experiments were performed on intentionally roughened LDH/*Si* substrates with R_q_ = 8 ∼ 40 nm achieved either by over depositing SL‐LDH or by spin coating 7 nm thick few layered (FL)‐LDH colloids onto *Si* wafers. The results revealed that the step barriers are effectively insurmountable for the ∼3 nm wide Pt branches (Figures S5 and S6). Instead of propagating across the interplate boundaries, Pt growth was confined within the initial LDH domains, yielding isolated 2D Pt nanoislands or hexagonal sub‐micron sized Pt sheets. In certain regions, pronounced out of plane overgrowth occurred, giving rise to three dimensional (3D) Pt dendrites preferentially at the periphery of the LDH plates, which further impeded continuous Pt propagation across the wafer scale area. Notably, the FL‐LDH/*Si* configuration, which exposes a larger reactive edge area due to the laterally fragmented LDH discs, promoted multidirectional 3D dendritic Pt growth at the LDH edges (Figure S6). This morphology prevents the lateral interconnection of 2D Pt layers between adjacent LDH plates and thus suppresses the formation of a continuous, wafer spanning den‐2DPt architecture. Collectively, these results demonstrate that the seamless propagation of ∼3 nm‐sized Pt domains across neighboring LDH template surfaces on an ultrasmooth f‐LDH/*Si* substrate (R_q_ = 1.15 nm) is essential for bridging nanoscale growth processes and desired wafer‐scale 2D metal architectures, thereby enabling the fabrication of continuous centimeter‐scale dendritic Pt films. X‐ray photoelectron spectroscopy (XPS) of the final *den‐2DPt_waf_*/f‐LDH/*Si* heterostructure revealed a dominant contribution from metallic Pt(0), with the Pt 4f core‐level peaks exhibiting a small but distinct shift of ∼0.3 eV toward lower binding energy relative to the standard Pt(0) reference (Figure S7). This negative binding‐energy shift is consistent with strong interfacial interaction between Pt and the f‐LDH surface, indicative of surface‐confined Pt growth and electronic coupling at the Pt–LDH interface.

### 2.3 Damage‐Free Transfer of the *den‐2DPt_waf_* on Various Substrates

We examined transfer of the *den‐2DPt_waf_* film onto diverse substrates. PMMA was adopted as a sacrificial support layer [43, 44]. The as‐grown *den‐2DPt_waf_* (1.0 cm^2^) film was first coated with PMMA (Figure 3a and Figure S8). The transfer involved removal of the SiO_x_ layer located between the *den‐2DPt_waf_*/f‐LDH and Si(100) substrate by applying HF solution, which liberated the *den‐2DPt_waf_* from the substrate (Figure 3b). In contrast, in the absence of the PMMA support layer, the *den‐2DPt_waf_* disintegrated into micron‐sized flakes (Figure S3). So, the laminated PMMA‐supported *den‐2DPt_waf_* film was subsequently transferred onto various target substrates; following the PMMA removal, intact and transparent *den‐2DPt_waf_* films with large lateral dimension, extending up to 5 × 5 cm^2^, were obtained on *Si* wafers (i), copper (Cu) foil (ii), glass (iii), carbon paper (iv), and flexible polyethylene terephthalate (PET) (Figure 3c,d and Figures S9–S11).

**FIGURE 3:**
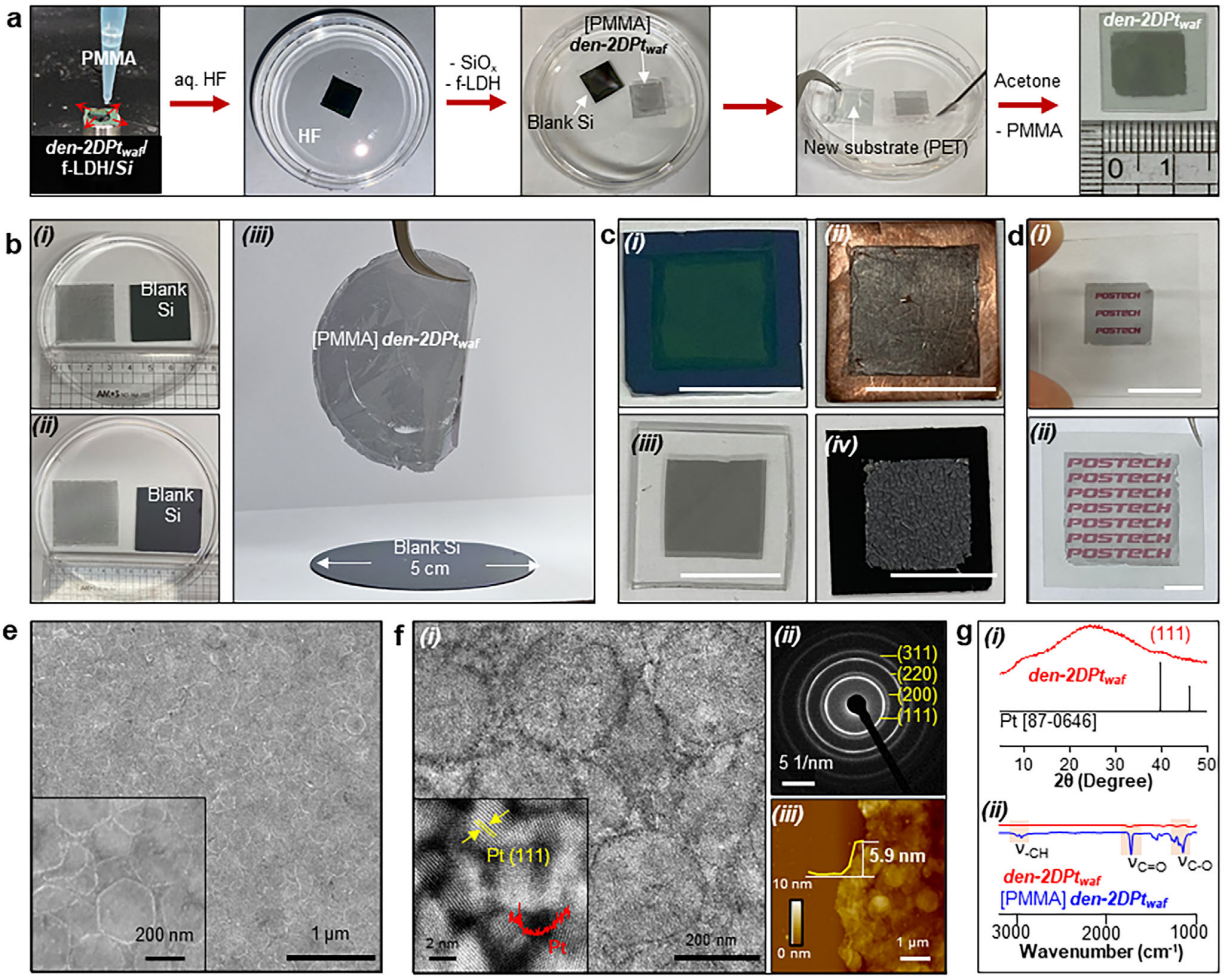
Scalable transfer and multi‐substrate integration of the *den‐2DPt_waf_* film. (a) Schematic presentation highlighting the steps for the transfer of the *den‐2DPt_waf_* [size = 1 cm × 1 cm] from the *den‐2DPt_waf_*/f‐LDH/*Si* into PET. (b) Digital images of free‐standing [PMMA]/*den‐2DPt_waf_* detached from Si wafer at different sizes [(3 cm × 3 cm) & (5 cm × 5 cm); (*i*), (*ii*)] and shape (circular shape, *iii*). (c) Digital images of 1.0 cm^2^‐sized *den‐2DPt_waf_* on various arbitrary substrates, like Si wafer (*i*), Cu foil (*ii*), glass (*iii*), and carbon paper (*iv*) without any mechanical damage to *den‐2DPt_waf_* film [scale bar = 1.0 cm]. (d) Digital images of the transparent *den‐2DPt_waf_* film on glass (*i*) and PET substrate (*ii*) [scale bar = 1 cm]. (e) SEM image at different magnifications, f, TEM image and high resolution high‐angle annular dark‐field (HAADF)‐STEM image with EDS‐line mapping (*i*), and SAED pattern (*ii*) of the *den‐2DPt_waf_* film, height‐profile AFM image on fresh Si wafer (*iii*). (g) XRD pattern (*i*) and FTIR graph (*ii*) for the *den‐2DPt_waf_* film transferred on glass.

Low magnification SEM and optical microscope images of the *den‐2DPt_waf_* film verified the reliability of the transfer protocol, showing a defect free, wrinkle free Pt nanolayer continuously spanning large lateral areas (Figure 3e and Figures S12–S14). Plane view TEM revealed that the film is constructed from laterally interconnected, hexagonal Pt discs that tile the entire *den‐2DPt_waf_* film. High‐resolution scanning transmission electron microscopy (STEM) further confirmed the preservation of the dendritic architecture, characterized by channel‐like nanoporosity with 3–5 nm gaps between Pt branches and a nearly uniform image contrast, consistent with a flat, homogeneous 2D Pt sheet (Figure 3f (i)). Corresponding energy‐dispersive x‐ray spectroscopy (EDS) mapping showed no detectable Ni or Co, consistent with the complete removal of the f‐LDH template during HF treatment (Figure S15). The measured lattice spacing of the Pt(111) planes (d ≈ 0.227 nm) evidenced a strain free metallic Pt phase in the *den‐2DPt_waf_* film. The selected‐area electron diffraction (SAED) pattern indicates a polycrystalline face centered cubic (fcc) Pt structure arising from multiple nucleation and subsequent lateral coalescence (Figure 3f (ii)). The average thickness of *den‐2DPt_waf_* measured by AFM was 5.9 nm (Figure 3f (iii)). X‐ray diffraction (XRD) of the transferred Pt layer exhibited only broad, low intensity diffraction features, which is characteristic of ultrathin metallic films (Figure 3g (i) and Figure S16). Fourier transform infrared (FTIR) spectra confirmed the complete removal of PMMA residues from the *den‐2DPt_waf_* film, ensuring a clean Pt surface favorable for surface sensitive applications (Figure 3g (ii)).

### 2.4 Electrochemical Characterization of the *den‐2DPt_waf_* Bioelectrode

The electrical conduction of the *den‐2DPt_waf_* film was evaluated in the lateral direction by transferring the *den‐2DPt_waf_* film onto an array of Au line patterns (Figure 4a). From the current measured between two Au lines overcoated with the *den‐2DPt_waf_* film, the *den‐2DPt_waf_* film was electrically insulating as numerous grain boundaries between its nanoscale crystalline domains impede continuous in‐plane charge transport [45]. This result demonstrates that the *den‐2DPt_waf_* film does not electrically bridge adjacent Au traces. The current values are presented as mean ± SD from five independently fabricated samples. The exposed geometric area of each electrode was independently defined by the underlying Au electrode pattern and PI passivation opening as 0.04 mm^2^. Accordingly, the electrochemical performance of the *den‐2DPt_waf_* was evaluated after transfer onto the Au electrode array. The detailed fabrication procedures of the Au electrodes are described in the Methods section and schematically illustrated in Figure S17, yielding electrodes with an active area of 0.04 mm^2^. For comparison, three electrode variants were prepared: (i) a bare Au electrode, (ii) a control electrode coated with a 6 nm‐thick sputtered Pt film (sp‐Pt/Au), and (iii) an electrode coated with an as‐transferred *den‐2DPt_waf_* film (*den‐2DPt_waf_*/Au; Pt thickness = 6 nm). Electrochemical characterization was subsequently performed in phosphate‐buffered saline (PBS; pH 7.4) using five electrodes fabricated from *den‐2DPt_waf_* film sections sampled from the same wafer, including center and edge regions. The measurements included electrochemical impedance spectroscopy (EIS), cyclic voltammetry (CV), and voltage transient measurements to determine impedance, CSC, and CIC.

**FIGURE 4:**
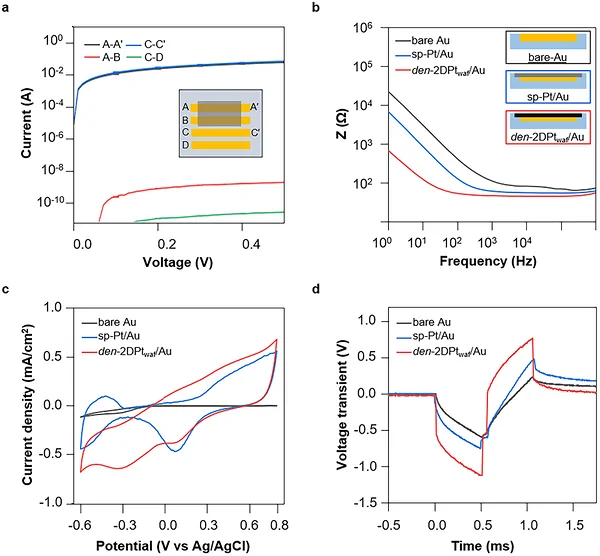
Electrochemical characterization of the *den‐2DPt_waf_*/Au electrode and control bioelectrodes. (a) *I*–*V* curves of *den‐2DPt_waf_*/Au circuit electrodes [inset: schematic showing Au pattern (yellow), *den‐2DPt_waf_* (grey), and PI substrate (blue)]. (b) EIS at 50 mV sinusoidal input, for bare Au, sp‐Pt/Au and *den‐2DPt_waf_*/Au (black, blue, and red, respectively). Inset: color‐matched schematics of the electrode structures used to identify the curves in (b–d). (c) CV at scan rate = 50 mV s^−1^, and (d) voltage transients under biphasic pulses (0.5 ms width, 0.05 ms interphase delay) recorded at the respective maximum charge‐injection currents of each electrode (0.9 mA for *den‐2DPt_waf_* (red), 0.7 mA for sp‐Pt/Au (blue), and 0.025 mA for bare Au (black)) in Ar‐saturated PBS (0.01 M, pH 7.4). Data in (a–d) were obtained from five independently fabricated electrodes (n = 5).

Figure 4b compares the EIS results, which revealed that the *den‐2DPt_waf_*/Au electrode exhibited markedly lower impedance than both bare Au and sp‐Pt/Au electrode across the full frequency range, with the most significant suppression at low frequencies. At 1 kHz and 1 Hz, *den‐2DPt_waf_*/Au electrode showed impedances of 45.4 ± 1.1 and 110.7 ± 6.56 Ω, reflecting significant decrease from the sp‐Pt/Au electrode (60.0 ± 2.11 Ω at 1 kHz and 852.2 ± 7.46 Ω at 1 Hz). This enhancement arises from the intrinsic dendritic architecture of *den‐2DPt_waf_*, in which the growth process naturally generates nanoscale gaps (∼3 nm) between adjacent Pt branches. The resulting interconnected nanoporosity provides additional electrochemically accessible electrode–electrolyte interfaces, contributing to the enhanced interfacial capacitance and electrochemical performance relative to the sputtered Pt control [46, 47]. Consistently, CV of the *den‐2DPt_waf_*/Au electrode exhibited broader voltametric envelopes and higher current responses than the other electrodes, yielding a maximum CSC of 11.97 ± 0.01 mC cm^−2^ (Figure 4c). Although the sp‐Pt/Au electrode substantially increased the CSC (8.45 ± 0.02 mC cm^−^^2^) compared with bare Au (0.30 ± 0.01 mC cm^−^^2^), its value remained lower than that of the *den‐2DPt_waf_*/Au electrode, reflecting the enhanced charge storage enabled by the dendritic nanoporous structure. Voltage transient measurements under biphasic current stimulation further showed that the *den‐2DPt_waf_*/Au electrode‐maintained voltage excursions well within the water window even at elevated current amplitudes (Figure 4d). This stable polarization behavior enabled a CIC of 1.13 mC cm^−2^, exceeding those of the sp‐Pt/Au (0.88 mC cm^−2^) and bare Au (0.031 mC cm^−2^). CIC was determined from the maximum discrete simulation‐current step that maintained the electrode potential within the water electrochemical window. All five *den‐2DPt_waf_*/Au electrodes reached the same current‐step threshold, and therefore no variation in CIC was observed among the five electrodes within the resolution of the stepwise measurement protocol. Such elevated CIC is particularly advantageous for neural stimulation, as it supports effective neuronal activation at reduced current thresholds, thereby minimizing energy demands while remaining within established electrochemical safety limits. Notably, the *den‐2DPt_waf_*/Au electrode exhibited highly reproducible electrochemical characteristics (Figure S18), and its overall performance compares favorably with previously reported Pt‐coated electrodes, underscoring its strong potential for advanced biointerface applications (Table S1).

### 2.5 Mechanical Robustness and Long‐Term Durability of the *den‐2DPt_waf_*/Au Electrode

Beyond electrochemical performance, the practical implementation of implantable bioelectrodes critically depends on mechanical robustness and long‐term chemical stability under physiologically relevant conditions. While the conventional PEDOT:PSS‐coated electrode can deliver relatively high CIC values (2.75 mC cm^−2^) due to the presence of a highly conductive polymer layer, their long‐term mechanical and interfacial stability remains an important consideration for chronic bioelectronic applications [48]. Although the PEDOT:PSS coating enhanced the CIC (Figure S19a), the layer was easily detached by gentle rubbing with a cotton swab (Figure S19b), indicating weak interfacial adhesion under the rubbing test. In contrast, the *den‐2DPt_waf_*/Au electrode remained intact under the same conditions, suggesting stable interfacial attachment to the underlying electrode structure.

The long‐term chemical stability of the electrodes was evaluated under harsh oxidative conditions relevant reactive oxygen species rich inflammatory environments. The electrodes were immersed in 3% H_2_O_2_ in PBS, which is an accelerated aging protocol, imposes oxidative stress on the electrode and its interface. Under these conditions, the *den‐2DPt_waf_*/Au electrode retained its structural integrity throughout the test, preserving a smooth, crack‐free morphology even after seven days of accelerated aging at 85°C, as confirmed by optical microscopy (OM) and SEM imaging (Figure 5a and Figures S20 and S21). This improved durability was related with the homogeneously distributed nanoporosity and intrinsic mechanical compliance of the dendritic Pt network, which could help accommodate local stress and facilitate gas release. However, sp‐Pt/Au underwent pronounced structural degradation even at room temperature, as evidenced by SEM images showing extensive surface cracking and delamination of the sputtered Pt film, while the corresponding EDS analyses revealed exposure of the underlying Au layer in the degraded regions, confirming loss of the Pt overlayer (Figure 5b and Figure S20). These failures are plausibly attributed to localized mechanical stresses arising from the catalytic H_2_O_2_ decomposition at the Pt surface, which produces oxygen bubbles at the electrode–electrolyte interface and induces interfacial delamination [49, 50].

**FIGURE 5:**
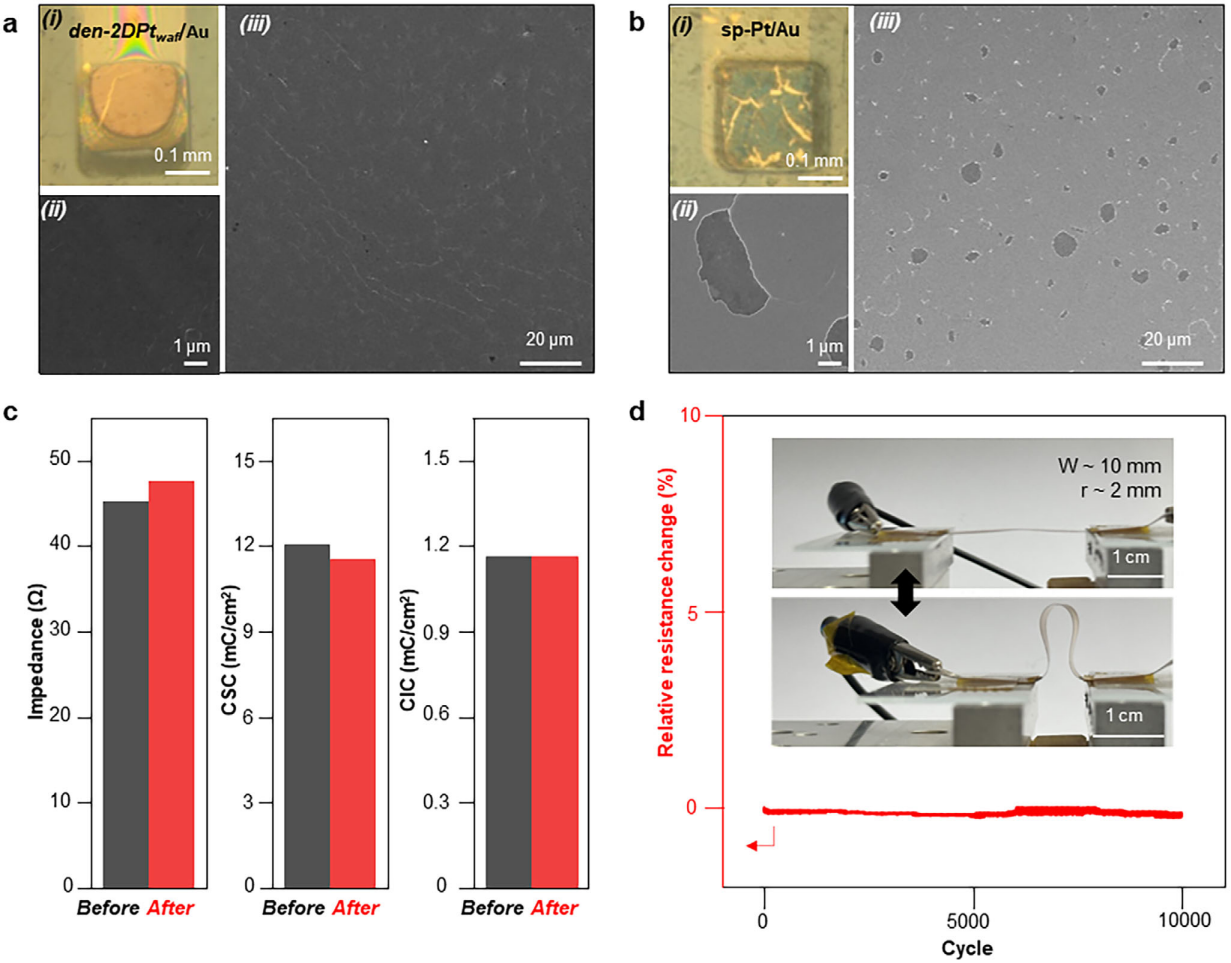
Chemical and mechanical resilience of the *den‐2DPt_waf_*/Au electrode and a control electrode made by sputtering Pt on Au (sp‐Pt/Au). (a,b) OM (*i*) and SEM (*ii, iii*) images of 0.04 mm^2^ electrodes after accelerated oxidative aging (PBS + H_2_O_2_, 85°C, 7 days). The *den‐2DPt_waf_*/Au electrode (a) retains its structural integrity and surface morphology after aging, whereas the sp‐Pt/Au electrode (b) shows pronounced degradation and delamination. (c) Electrochemical performance measured before (gray) and after (red) aging for *den‐2DPt_waf_*/Au electrode, including impedance (at 1 kHz) (left), CSC (middle), and CIC (right). (d) Relative resistance changes of the *den‐2DPt_waf_*/Au electrode during cyclic bending (10 000 cycles, bending radius ≈ 2 mm); Inset: optical image of the bending setup (specimen width W ≈ 10 mm).

Consistent with the morphological observations, electrochemical characterization showed that the *den‐2DPt_waf_*/Au electrode exhibited only marginal changes in impedance and CSC (5.2% and 4.4%, respectively) over the testing period, while CIC remained essentially unchanged (Figure 5c and Figure S22). By contrast, the sp‐Pt/Au electrode resulted in electrical discontinuity (Figure S23). These observations establish the *den‐2DPt_waf_/*Au electrode as a highly resilient bioelectrode capable of sustaining long‐term operation under oxidative physiological conditions, with reproducibility tests further confirming its excellent structural stability and showing no noticeable delamination or structural damage after oxidative stress exposure (Figure S24). To further evaluate the long‐term electrochemical stability under repeated neural stimulation, the *den‐2DPt_waf_*/Au electrodes were subjected to repetitive biphasic electrical pulse stimulation in a simulated physiological environment. (Figure S25) The EIS and CV characteristics measured before and after 50 000 biphasic stimulation pulses showed overlapping profiles, with only a 5.66% increase in impedance at 1 kHz and a 4.75% decrease in CSC. These results indicate that the *den‐2DPt_waf_*/Au electrode maintains its electrochemical characteristics even after extensive repetitive electrical stimulation, demonstrating cycling stability for neural stimulation applications. The mechanical flexibility of the electrode was examined by monitoring resistance variations during repeated bending. Because wire segments experience the highest mechanical strain during device operation, durability tests were conducted on Au lines patterned on PI rather than on the electrode pads. Notably, the *den‐2DPt_waf_*/Au electrode maintained resistance fluctuations below 0.05% after 10,000 bending cycles at a bending radius of 5 mm (Figure 5d), demonstrating good stability against mechanical deformation under conditions directly relevant to flexible and implantable bioelectronic systems.

Representative hematoxylin‐and‐eosin (H&E)‐stained sections from the implanted and sham‐operated animals are presented in Figure S26. No dense neutrophilic infiltration indicative of a pronounced ongoing acute inflammatory response was apparent in the examined sections of the heart, liver, lung, kidney or spleen in either animal. Similarly, neither the immediate peri‐electrode tissue from the implanted animal nor the corresponding implant‐pocket tissue from the sham animal exhibited conspicuous neutrophil‐rich inflammatory accumulation in the examined sections. These qualitative observations provide preliminary evidence that the proposed electrode did not induce marked ongoing acute inflammation at the 2‐week endpoint.

### 2.6 Vagus Nerve Stimulation (VNS) With the *den‐2DPt_waf_*/Au Electrode

To further verify the stimulation capability of the *den‐2DPt_waf_*/Au electrode, real‐time physiological responses to VNS were recorded, as schematically depicted in Figure 6a. A step‐up stimulation paradigm was employed in which the current amplitude was increased in 100 µA increments while all other stimulation parameters were held constant. Throughout the stimulation sequence, photoplethysmography (PPG) signals were continuously acquired and digitally filtered to isolate the respiratory component, enabling quantitative extraction of the respiratory rate (breaths per minute, BPM). As the stimulation current increased, a stepwise reduction in respiratory rate was observed, which is consistent with graded functional engagement of vagal fibers reported in previous VNS studies [51].

**FIGURE 6:**
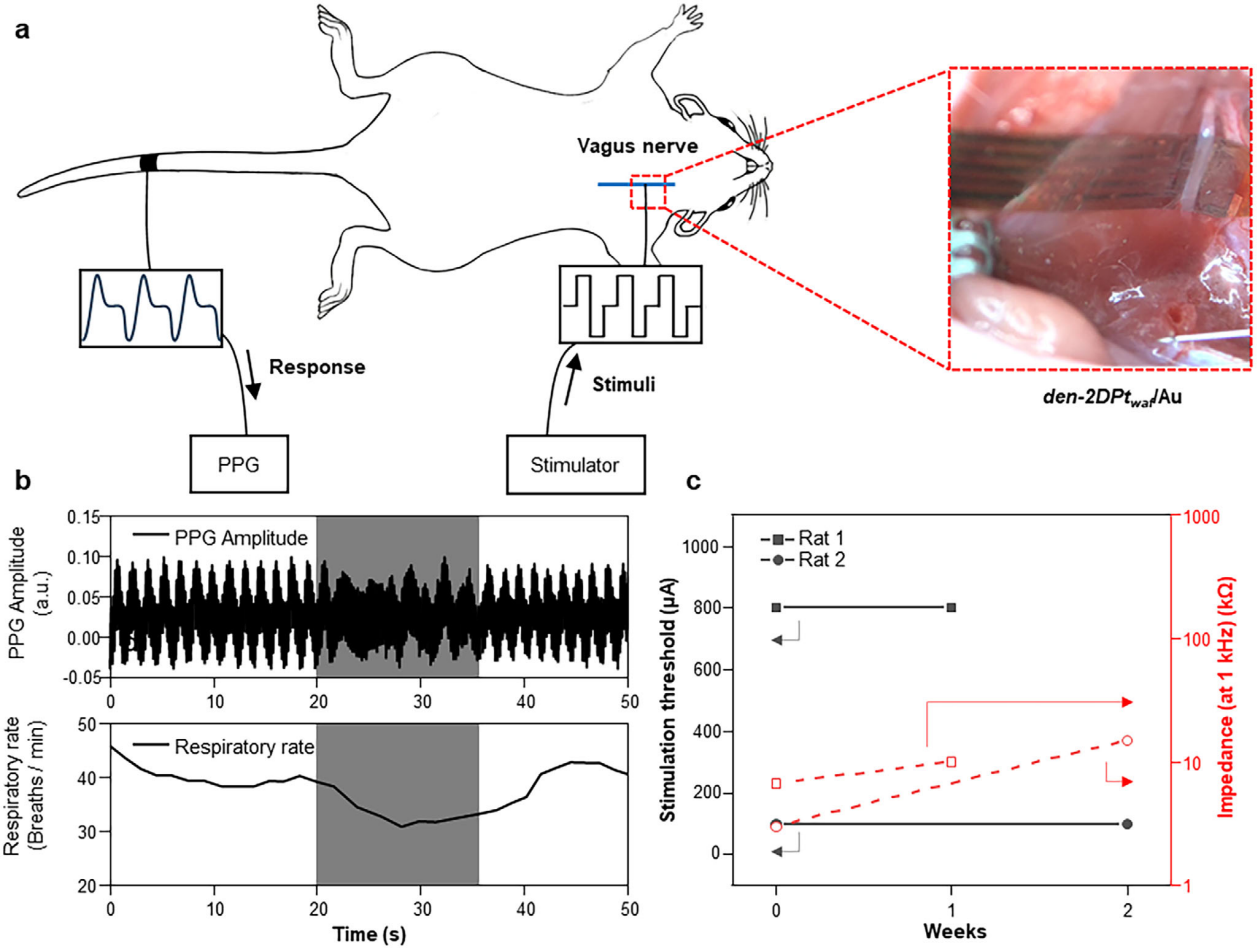
In vivo validation of vagus nerve stimulation (VNS) by using the *den‐2DPt_waf_*/Au electrode. (a) Schematic illustration of the in vivo VNS setup. The *den‐2DPt_waf_*/Au electrode was implanted onto the rat vagus nerve, and electrical stimulation was applied while physiological responses were monitored via PPG to extract respiratory rate. The red dashed box corresponds to the inset digital image, highlighting the mechanical adaptability of the *den‐2DPt_waf_*/Au electrode interfaced with the vagus nerve in vivo. (b) Representative PPG‐derived respiratory waveform recorded at the functional stimulation threshold 2 weeks post‐implantation, showing effective physiological modulation during electrical stimulation. The gray region indicates the stimulation period. (c) Time‐dependent stability of the functional stimulation threshold (filled black symbols, left axis) and electrochemical impedance at 1 kHz (open red symbols, right axis) measured in independent rat cohorts evaluated at each post‐implantation time point, demonstrating stable electrochemical characteristics and reliable in vivo stimulation performance of the *den‐2DPt_waf_*/Au electrode.

To evaluate electrode function while minimizing physiological stress associated with repeated surgeries, separate individual animals were evaluated at week 0, week 1, and week 2. The functional stimulation threshold was defined as the minimum current that reproducibly elicited respiratory modulation, and its robustness was confirmed by consistent stimulus–response relationships across the applied current range. Below this threshold, no discernible change in respiratory rate was detected, whereas stimulation at or above threshold reliably induced respiratory modulation. This graded response was consistently observed in the individual animals evaluated at week 0, week 1, and week 2, (Figures S27 and S28), indicating that the identified threshold reliably marks the onset of physiological response to VNS over time and that the *den*‐*2DPt_waf_*/Au electrode maintains a consistent stimulation response during subacute implantation.

Figure 6b shows a representative PPG‐derived respiratory waveform recorded at the functional stimulation threshold two weeks after implantation. At this subacute time point, vagus nerve stimulation produced clear and reproducible modulation of the respiratory rate, confirming robust functional coupling between electrical stimulation and physiological output under stable implantation conditions. Figure 6c summarizes the temporal evolution of both the functional stimulation threshold and the electrochemical impedance of the implanted electrodes. The stimulation thresholds measured in the individual animals were comparable, and relatively low impedance values were observed at each evaluated time point. However, because only one animal was evaluated each week, these observations don't establish animal reproducibility. In line with these functional data, visual inspection of the explanted devices revealed no apparent mechanical damage, delamination, or structural failure (Figure S29). However, detailed high‐magnification structural analysis of the Pt surface was obstructed by the residual tissues adhered to the *den*‐*2DPt_waf_*/Au electrode (Figure S30). All stimulation currents applied in vivo were kept well below the CIC of the *den*‐*2DPt_waf_*/Au electrode (1.13 mC cm^−2^), corresponding to injected charge densities of 0.05 mC cm^−2^ at 100 µA and 0.4 mC cm^−2^ at 800 µA, thereby ensuring operation within the established electrochemical safety limits. All stimulation currents applied in vivo were kept well below the CIC of the *den‐2DPt_waf_*/Au electrode (1.13 mC cm^−2^), corresponding to injected charge densities of 0.05 mC cm^−2^ at 100 µA and 0.4 mC cm^−2^ at 800 µA, thereby ensuring operation within the established electrochemical safety limits.

## 3 Conclusion

We report a wafer‐scale synthesis of a continuous ultrathin nanoporous Pt film (*den‐2DPt_waf_*) consisting of dendritic 2D Pt nanosheets via surface‐confined Pt growth on LDH‐modified Si wafers. Controlled roughness of the LDH‐modified *Si* wafer promotes barrier‐free lateral propagation of Pt. Clean transfer of the *den‐2DPt_waf_* film onto diverse substrates circumvents the constraints of direct metal growth on incompatible surfaces. When used as a bioelectrode, the stacked architecture of flexible nanosheets renders the *den‐2DPt_waf_* film vertically conductive but laterally insulating; consequently, the electrochemically active area of the *den‐2DPt_waf_* film transferred onto patterned Au is defined by the area of the Au pattern. The dendritic nanoporosity of the *den‐2DPtwaf* electrode boosts charge storage and injection capacity, enabling efficient neural stimulation within safe electrochemical limits. In addition, in vivo VNS experiments demonstrated functional respiratory modulation in individual animals evaluated at week 0, week 1, and week 2 after implantation. However, only one animal was evaluated at each time point. Further studies with larger animal cohorts and longitudinal evaluation are required to establish long‐term in vivo stability and reproducibility. Moreover, although qualitative mechanical tests indicated stable attachment of the *den‐2DPtwaf* film, the interfacial bonding strength with the Au/PI substrate was not quantitatively determined. Future studies using nano‐scratch or micro‐mechanical testing will be required to quantify the interfacial adhesion strength. Although the wafer‐scale fabrication process enables scalable production of *den‐2DPtwaf* electrodes, the current transfer‐based integration step limits the achievable pattern resolution. Further optimization of the transfer and patterning processes will therefore be required to realize high‐resolution multichannel neural interfaces. Collectively, these results establish *den‐2DPtwaf* as a mechanically compliant, highly conductive, and electrochemically efficient platform for neural stimulation and recording interfaces.

## 4 Experimental Section/Methods

### 4.1 Reagents

All reagents, including nickel(II) nitrate hexahydrate (Thermo, A15540), cobalt(II) nitrate hexahydrate (Aldrich, 239267), hexamethylenetetramine (HMT; Acros, 120610010), sodium dodecyl sulfate (SDS; Thermo, 230421000), formamide (Samchun Chem., 000F0325), sodium tetrachloroplatinate(II) hydrate (Strem, 93–7810), L(+)‐ascorbic acid (Samchun Chem., A1182), PVP (Aldrich, 102744463), PMMA (Aldrich, 1003394966), chlorobenzene (Thermo, 396970010), hydrofluoric acid (Samchun Chem., 49.0%, 000H1457), sulfuric acid (Samchun Chem., 95.0%, 000S4750) and potassium hydroxide (Samchun Chem., 95.0%, P1446), sodium hydroxide (Samchun Chem, 98.0%, S0610), PI (P84; Evonik), H_2_O_2_ (Aldrich, 35%, 1.08600), 1× phosphate buffer saline (PBS; pH 7.4, Aldrich, P4474), poly(acrylic acid) (M.W.∼2000, Aldrich, 535931) were used as received without further purification. A silicon wafer with a SiO_x_ coating (thickness = 500 ± 25 nm) was purchased from Namgang Hightech. Other substrates such as carbon fiber paper (Fuel Cell Store, 39BB, thickness = 315 ± 20 µm, 63070045), indium tin oxide coated–PET (ITO‐PET) sheet (TECHINSTRO, thickness = 0.175 mm, CL‐TE001), FTO‐coated glass (Aldrich, thickness = 2.2 mm, 735167), copper foil (Alfa Aesar, 99.9%, thickness = 0.254 mm, 013379), glass slides (thickness = 1.1 ± 0.1 mm, M2629) and titanium fiber felt (Bekaert, thickness = 0.2 mm, Currento PTL Ti‐67/200) were thoroughly cleaned and washed before use. Throughout the experiments, deionized (DI) water was used unless stated otherwise.

### 4.2 Synthesis of f‐LDH/Si

SL‐LDH (lateral size = 262 ± 28 nm) were synthesized according to a reported method [52]. Briefly, an aqueous mixture containing nickel(II) nitrate (0.5 M), cobalt(II) nitrate (0.3 M), SDS (0.25 M), and HMT (1 M) was hydrothermally treated at 120°C for 24 h. The resulting precipitate was collected, washed with water and ethanol, and dried. To obtain single‐layer nanosheets, the precipitate (50 mg) was exfoliated in formamide (50 mL) at 40°C for several days, followed by centrifugation (2000 rpm); the supernatant was collected as the SL‐LDH nanosheet dispersion. The SL‐LDH nanosheets were subsequently isolated by centrifugation and redispersed in ethanol. Then, as‐prepared ethanolic SL‐LDH dispersion (0.01 mL, 20 mg mL^−^^1^) was spin‐coated onto thoroughly cleaned SiO_x_/Si(100) substrates (i.e., *Si*; first standardized area = 1 cm × 1 cm). The native SiO_x_ layer was utilized to improve substrate hydrophilicity and enhance adhesion of the SL‐LDH layers. Following solvent evaporation, the SL‐LDH nanosheets spontaneously assembled into a continuous thin film across the substrate surface and were subsequently dried at room temperature. Spin‐coating parameters, including SL‐LDH concentration, deposited volume, and rotation speed (optimized at 6000 rpm for 60 s), were systematically varied to obtain smooth and fully covered f‐LDH films on *Si*.

### 4.3 Synthesis of *den‐2DPt_waf_/f‐LDH*

The f‐LDH/*Si* substrate (1 × 1 cm^2^) was immersed in 2.0 mL of an aqueous Pt precursor solution (Na_2_PtCl_4_·xH_2_O, 1.25 mM) at room temperature for 30 min. Subsequently, 1.0 mL of ascorbic acid (6.7 mM) and 1.0 mL of PVP (MW ≈ 55 000, 45 mg mL^−1^) were immediately added, and the resulting reaction mixture (pH ≈ 3.5) was placed in a preheated oil bath at 70°C for 30 min to grow Pt, yielding the *den‐2DPt_waf_*/f‐LDH. After growth, the substrate was removed and rinsed with deionized water and ethanol. The sacrificial SiO_x_ layer was subsequently removed by etching with 5% HF (10 min, room tem.), which also dissolved the f‐LDH layer and thereby released a centimeter‐scale free‐standing *den‐2DPt_waf_* film from the substrate for subsequent characterization. Once the synthetic protocol for the *den‐2DPt_waf_*/f‐LDH/*Si* on a 1 × 1 cm^2^ wafer was established, the lateral dimensions were readily scaled up to 5 × 5 cm^2^, and to other wafer geometries (e.g., circular *Si*), by proportionally increasing the precursor quantities according to the wafer area while maintaining identical reaction conditions (Table S2).

### 4.4 Transfer of the *den‐2DPt_waf_* Onto Various Substrates

The wet‐transfer for the *den‐2DPt_waf_* employed PMMA as a support layer spin‐coated on the *den‐2DPt_waf_*/f‐LDH, followed by selective SiO_x_ etching in an aqueous HF solution in order to release the PMMA‐supported *den‐2DPt_waf_*. Typically, a PMMA solution (MW ≈ 996 000; 80 mg mL^−1^ in chlorobenzene) was spin‐coated onto the *den‐2DPt_waf_*/f‐LDH/*Si* (1.0 cm^2^ wafer, 0.03 mL solution; 4000 rpm, 60 s) and baked at 120°C for 30 min. The resulting [PMMA]/*den‐2DPt_waf_*/f‐LDH/*Si* stack was immersed in 5% HF solution for 30 min to dissolve the f‐LDH and SiO_x_ layers, thereby fully releasing the *den‐2DPt_waf_* film from the wafer. The etchant was then repeatedly exchanged with DI water to rinse the floating [PMMA]/*den‐2DPt_waf_* film and minimize residual corrosive species. While the [PMMA]/*den‐2DPt_waf_* film remained at the air–water interface, fresh substrates (e.g., Si wafers, carbon paper, Cu foil, PET) were brought into contact or gently scooped up with tweezers to pick up the film, followed by vacuum drying for several hours to enhance adhesion and remove residual solvents. Finally, the PMMA support was removed by soaking the [PMMA]/*den‐2DPt_waf_* in acetone for 15 min and air‐drying for several hours, yielding the transferred *den‐2DPt_waf_* film on the target substrate. The target substates include gas diffusion layers (GDLs), flexible polymer films, and metallic Cu/Ti foils. Prior to transfer, slide glass, FTO‐coated glass, Ti fiber felt, and Cu foil were rigorously cleaned by sequential rinsing with deionized (DI) water and ultrasonication in acetone and DI water (5 min each). ITO‐PET was ultrasonically cleaned in DI water and ethanol (3 min each) and then rinsed with DI water.

### 4.5 Fabrication of the *den‐2DPt_waf_*/Au Electrode

A 4‐inch Si wafer was sequentially cleaned with acetone, isopropanol, and deionized water. A sacrificial layer was then formed by spin‐coating a 10 wt.% poly(acrylic acid) solution at 3000 rpm for 30 s. Subsequently, a PI layer was spin‐coated, followed by soft‐baking at 80°C and hard‐baking at 250°C. Au patterns were defined via thermal evaporation through a metal shadow mask, after which a thin PI overlayer was spin‐coated and baked for encapsulation. Reactive ion etching (RIE) was employed to selectively open Au pad and wire regions (250 W, 5 min, 100 sccm O_2_, 10 sccm CHF_3_). The polyimide (PI) film was etched using a RIE system (VITA‐E, Femto Science Inc.) at an RF power of 250 W and an O_2_ flow rate of 100 sccm. The *den‐2DPt_waf_* was then transferred onto the patterned substrate. To release the devices, the wafer was immersed in water to dissolve the poly(acrylic acid) sacrificial layer. Finally, the electrodes were bonded to flexible cables by using a stretchable anisotropic conductive film (ACF) which was cured under the heat [53]. For the sp‐Pt/Au control, a Pt layer was sputter‐deposited through a metal shadow mask to confine the Pt coating to the predefined Au electrode area.

### 4.6 Electrical Property Characterization for the *den‐2DPt_waf_*/Au Electrode

EIS and CV were conducted in PBS using a PalmSens4 potentiostat. The fabricated electrodes were used as working electrodes (active surface area = 0.02 cm × 0.02 cm), with a Pt coil and Ag/AgCl serving as counter and reference electrodes, respectively. For EIS, a 50 mV sinusoidal excitation was applied, and all CV curves were acquired at a scan rate of 50 mV s^−1^. Before CV measurements, the PBS solution was purged with Ar for at least 30 min to remove dissolved oxygen. CIC was assessed using an Intan RHS stimulator system and a TBS2000 (Tektronix) oscilloscope by applying biphasic current pulses (phase width 0.5 ms, interphase delay 0.05 ms) and analyzing the resulting voltage transients to determine the maximum injectable charge within the water window. CSC and CIC were normalized to the exposed geometric electrode area of 0.04 mm^2^ for all electrode types.

### 4.7 Mechanical Endurance and Aging Tests

Cyclic bending tests were performed using a universal measurement system (UMP 100, Tera Leader Co. Ltd., Korea). To evaluate the mechanical flexibility and durability of the electrodes, repeated bending was conducted at a curvature radius of 2 mm and a frequency of 1 Hz for up to 10 000 cycles, while continuously monitoring the electrical resistance to detect any structural or electrical degradation. The chemical stability of the electrodes was further examined by immersing *den‐2DPt_waf_*/Au and sp‐Pt/Au electrodes in PBS containing 3wt.% H_2_O_2_ at 85°C for 7 days, that approximately corresponds to 224 days under physiological conditions [54]. This oxidative environment was employed to mimic reactive oxygen species (ROS)‐rich conditions associated with subacute implantation, thereby enabling assessment of the electrochemical and morphological stability of the electrodes under biologically relevant oxidative stress.

### 4.8 Histological Assessment of the Subacute Tissue Response

At 2 weeks after surgery, tissue specimens were collected from the heart, liver, lung, kidney, spleen, and the local implantation site from a total of two animals: one implanted animal and one sham‐operated animal (implant, n = 1; sham, n = 1). The sham animal underwent the same surgical incision and formation of the subcutaneous implant pocket as the implanted animal but did not receive an electrode. For the implanted animal, the implantation‐site specimen included the immediate peri‐electrode subcutaneous tissue adjacent to the salivary gland. The major‐organ histological panel was selected with reference to a previously reported assessment of an implantable neural electrode [55]. The collected tissues were processed for H&E staining and imaged at 10× and 20× objective magnification. Histological findings were assessed qualitatively, with particular attention to dense neutrophilic infiltration indicative of a marked ongoing acute inflammatory response.

### 4.9 Electrode Placement Surgery

Male Sprague Dawley rats (RRID:RGD_737903; 250–350 g, 8 weeks old; Orient Bio Inc., Republic of Korea) were used in this study. The implantation of flexible electrodes for VNS was performed following standard procedures described in previous literature [56]. Rat was anesthetized with inhalation isoflurane (5% in O_2_ at a flow rate of 0.4–0.6 L/min). After induction, a cervical incision was made to expose the left cervical vagus nerve, and the surgical field was prepared for electrode placement. The *den‐2DPt_waf_*/Au electrodes were gently positioned beneath the isolated vagus nerve to maintain close apposition without excessive mechanical compression. A stainless‐steel ground electrode was inserted into the nearby cervical muscle tissue. Following electrode implantation, the isoflurane concentration was maintained between 1∼2% throughout the procedure.

To assess physiological responses to VNS and PPG signals were recorded concurrently using a cuff‐type sensor (BIOPAC Systems Inc., Goleta, CA, USA) secured around the rat's tail. Data acquisition was continuous across baseline, stimulation, and post‐stimulation periods. VNS efficacy was evaluated using heart rate and respiratory rate as biomarkers, specifically looking for bradycardia and respiratory slowing (bradypnea or apnea) as indicators of vagus nerve engagement. Offline data processing was performed using custom Python 3.11 scripts (utilizing SciPy, NumPy, and pandas). Heart rate (BPM) was derived via peak detection on filtered signals, and respiratory rate was estimated from the PPG waveform's low‐frequency envelope

### 4.10 Vagus Nerve stimulation (VNS) With the *den‐2DPt_waf_*/Au Electrode

VNS was delivered using an Intan RHX system (Intan Technologies, Los Angeles, CA, USA). Prior to stimulation, electrode impedance was measured at 1 kHz using the system's integrated function to confirm electrical integrity. Stimulation consisted of charge‐balanced biphasic current pulses at 20 Hz for 15 s. Each pulse had a width of 200 µs with a 100 µs interphase delay. The stimulation amplitude was initially set to 50 µA and increased in 50 µA increments to identify the functional stimulation threshold, defined as detectable changes in heart rate and/or respiratory rate. If no functional response was observed after at least three attempts at a given amplitude, the step size was doubled. A 5 min resting period was provided between trials to minimize carryover effects. To assess post‐implantation electrode, three separate individual animals were evaluated at week 0, week 1, and week 2 after implantation, respectively. The same stimulation protocol was applied at each evaluation time point. The electrode was implanted for each cohort and maintained until the designated post‐implantation time point, and the same stimulation protocol was applied at week 0, week 1, or week 2 after implantation.

## Author Contributions

**Kyeongbin Kang**: investigation, writing – original draft, data curation, visualization, methodology. **Joosung Oh**: data curation, investigation, methodology, visualization, writing – original draft. **Soumen Dutta**: data curation, writing – original draft, conceptualization, writing – review and editing, visualization. **Sun Woo Jang**: conceptualization, investigation, methodology. **Byeong Su Gu**: investigation. **Woosung Cho**: investigation. **Mingyu Kwak**: investigation. **Sunguk Hong**: investigation. **Seongjae Lee**: investigation. **Hyo Kyoung Jeon**: investigation. **Juwan Han**: investigation. **Keehoon Kim**: supervision, methodology. **Sung‐Min Park**: methodology, supervision, writing – review and editing, funding acquisition. **Unyong Jeong**: conceptualization, supervision, writing – review and editing, funding acquisition, project administration, resources. **In Su Lee**: conceptualization, funding acquisition, writing – review and editing, supervision, resources, project administration.

## Ethics Statement

All animal experiments described in this study, including electrode implantation, vagus nerve stimulation, endpoint tissue collection, and histological assessment, were covered by the approval of the Institutional Animal Care and Use Committee of Pohang University of Science and Technology (POSTECH‐2025‐0099). All procedures were performed in accordance with the National Institutes of Health guidelines. The only biological resource used in this study was the Sprague–Dawley rat model (RRID:RGD_737903), specified at first mention in Section 4.9. No cell lines, antibodies, or other biological resources requiring RRIDs were used.

## Conflicts of Interest

The authors declare no conflicts of interest.

## Supporting information




**Supporting File**: advs78193‐sup‐0001‐SuppMat.pdf.

## Data Availability

The data that support the findings of this study are available from the corresponding author upon reasonable request.
