# Supplementary material for: Wafer‐Scale Dendritic Nanoporous Platinum Films for Neural Electrodes
Source: Adv Sci (Weinh). 2026 Oct 6:e78193. Online ahead of print. doi: 10.1002/advs.78193 (PMC13643091; doi:10.1002/advs.78193)
Supplement: Supplementary file 1 [file ADVS-9999-e78193-s001.pdf]

Supporting Information

Wafer-Scale Dendritic Nanoporous Platinum Films for Neural Electrodes

*Kyeongbin Kang,[1,2,†] Joosung Oh,[4,†] Soumen Dutta,[1,3,†] Sun Woo Jang,[1,3] Byeong Su Gu,[1,3] Woosung Cho,[4] Mingyu Kwak,[4] Sunguk Hong,[5] Seongjae Lee,[6] Hyo Kyoung Jeon,[7] Juwan Han,[5] Keehoon Kim,[5] Sung-Min Park,[*5,6] Unyong Jeong,[*4] In Su Lee[*1,2,3]*

[1]Center for Nanospace-confined Chemical Reactions (NCCR), Pohang University of Science and Technology (POSTECH), Pohang 37673 (Korea)

[2]Division of Advanced Materials Science, Pohang University of Science and Technology (POSTECH), Pohang 37673 (Korea)

[3]Department of Chemistry, Pohang University of Science and Technology (POSTECH), Pohang 37673 (Korea)

[4]Department of Materials Science and Engineering, Pohang University of Science and Technology (POSTECH), Pohang 37673 (Korea)

[5]Department of Mechanical Engineering, Pohang University of Science and Technology (POSTECH), Pohang 37673 (Korea)

[6]Major of Medical Science and Engineering, Pohang University of Science and Technology (POSTECH), Pohang 37673 (Korea)

[7]AX Graduate School, Pohang University of Science and Technology (POSTECH), Pohang 37673 (Korea)

[†]These authors contributed equally

[*]Correspondence authors E-mail: sungminpark@postech.ac.kr (S.-M. P.), ujeong@postech.ac.kr (U. J.), insulee97@postech.ac.kr (I. S. L.)

Keywords: two-dimensional Pt, wafer-scale synthesis, metallic nanofilm, resilient bio-interface, implantable bioelectrode

1. Materials Characterization.

1.1 Instruments.

Scanning electron microscopy (SEM) was carried out with HITACHI S-4800 (HITACHI) instrument. Transmission electron microscope (TEM) and High-resolution TEM (HRTEM) analyses were conducted with a JEOL JEM-2100 and JEM-2200FS with Image Cs-corrector at 200 kV. Atomic scale HAADF-STEM analysis was performed using a 200 kV operated STEM(ARM 200F, JEOL, Japan) with a spherical aberration corrector (ASCOR, CEOS GmbH, Germany) at Materials Imaging & Analysis Center of POSTECH in South Korea. For HAADF imaging, the camera length was to 10 cm with inner and outer detector angles ranging between 45-180 mrad and prob size of 9C. Scanning transmission electron microscope and energy-dispersive X-ray spectrometry (STEM-EDS) elemental mapping and line profiling were carried out using JEOL JEM-2100F at 200 kV. X-ray diffraction (XRD) patterns were recorded using a Miniflex (Rigaku) diffractometer with Cu-Kα radiation (λ-0.15418 nm) at 40 kV and 100 mA with a scan rate of 2θ = 4° min-1. Optical microscopic (OM) images were recorded in ECLIPSE Ts2 (Nikon). Inductively coupled plasma mass spectroscopy (ICP-MS): The concentrations of metal contents in the fabricated sample or electrolyte were measured with the ICP-MS (PerkinElmer, NexION 300s). Magnetic resonance (NMR) spectra were recorded on a Bruker 300 MHz and 500 MHz (AVANCE III Ascend 500, Broad-scope Nuclear Magnetic Resonance (NMR) Analysis Center supported by the Korea Basic Science, NFEC-2025-04-305346). The metal contents were determined by inductively coupled plasma atomic emission spectrometry (ICP-AES) using iCAP 7400 (Thermo ScientificTM). The oxidation state of the samples was determined using an X-ray photoelectron spectrometer (XPS) with an X-ray source of monochromated Al Kα (1486.6 eV) using a theta probe AR-XPS System (ThermoFisher, U.K.). Thickness of all of the synthesized samples was estimated from an atomic force microscope (AFM) instrument (Park Systems, Park NX20). The height-profile of the AFM images were obtained in a non-contact mode using Si-N tips with a nominal tip radium of<10 nm. (ACT part). Fourier transform infrared spectroscopy (FT-IR) of the samples were recorded in PerkinElmer instrument. Field emission scanning electron microscopy (FE-SEM, JEOL JSM-7401F) combined with energy-dispersive X-ray spectroscopy (EDS) was employed to analyze the surface morphology and elemental composition of the electrodes. In addition, the electrode surfaces were observed using optical microscopy (OM, BX51M, Olympus) to examine the overall geometry and surface uniformity.

1.2. Sample preparation for TEM, HRTEM and STEM-EDS.

The as-synthesized 2D metallic sheets were first separated from the Si substrate using an etchant solution to remove sacrificial SiO_x layer in between the product and silicon substrate. After this, the floated metallic films, such as *den-2DPt*$_{waf}$, *den-2DPt*$_{waf}$/f-LDH and others, were washed at

the air-liquid interface. Subsequently, they were laid on a carbon mesh Cu TEM grid for analysis after air-drying.

1.3. Sample preparation for XRD.

As-synthesized f-LDH and *den-2DPt*$_{waf}$/f-LDH samples were directly characterized through XRD following their synthesis and essential washings, while thin metallic film of transferred *den-2DPt*$_{waf}$ on a glass substrate was recorded for XRD as well.

1.4. Sample preparation for SEM, AFM and XPS.

Mostly, the as-synthesized products grown on *Si* substrate were directly checked for these studies, especially for SEM analyses. In contrast, transferred *den-2DPt*$_{waf}$ films on new *Si* wafer (thickness = 525 μm) were utilized for AFM and XPS analyses. Before use, wafers were cut into 1 cm × 1 cm followed by cleaning using acetone through sonication for 30 min.

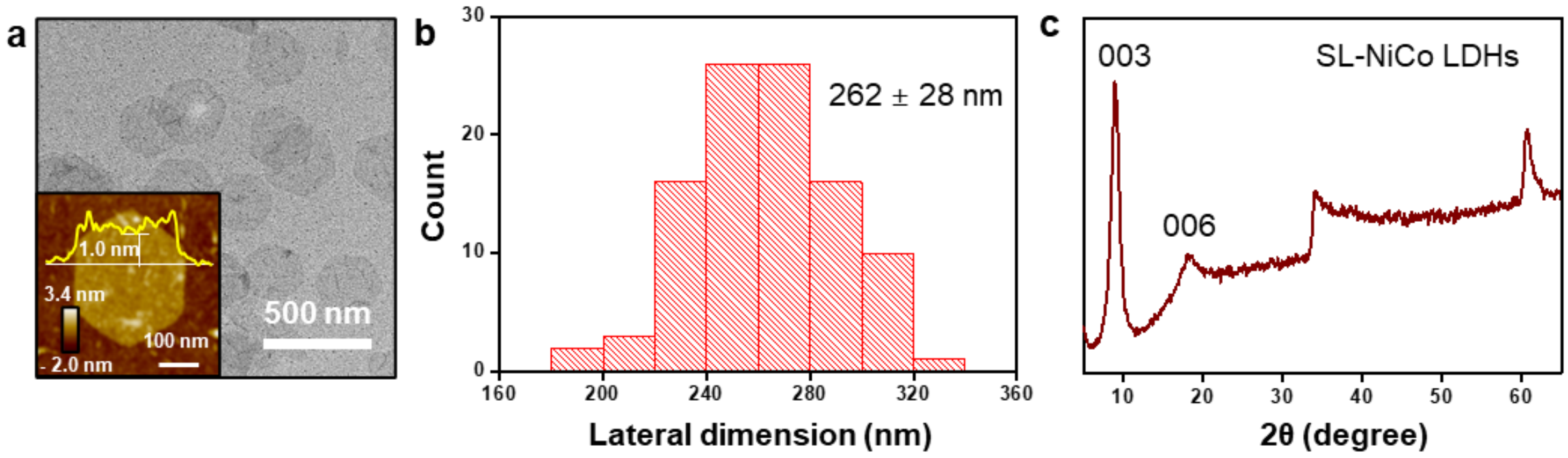

Figure S1. (a) TEM image (inset: AFM height profile), (b) size distribution plot, and (c) powder XRD pattern for SL-LDHs.

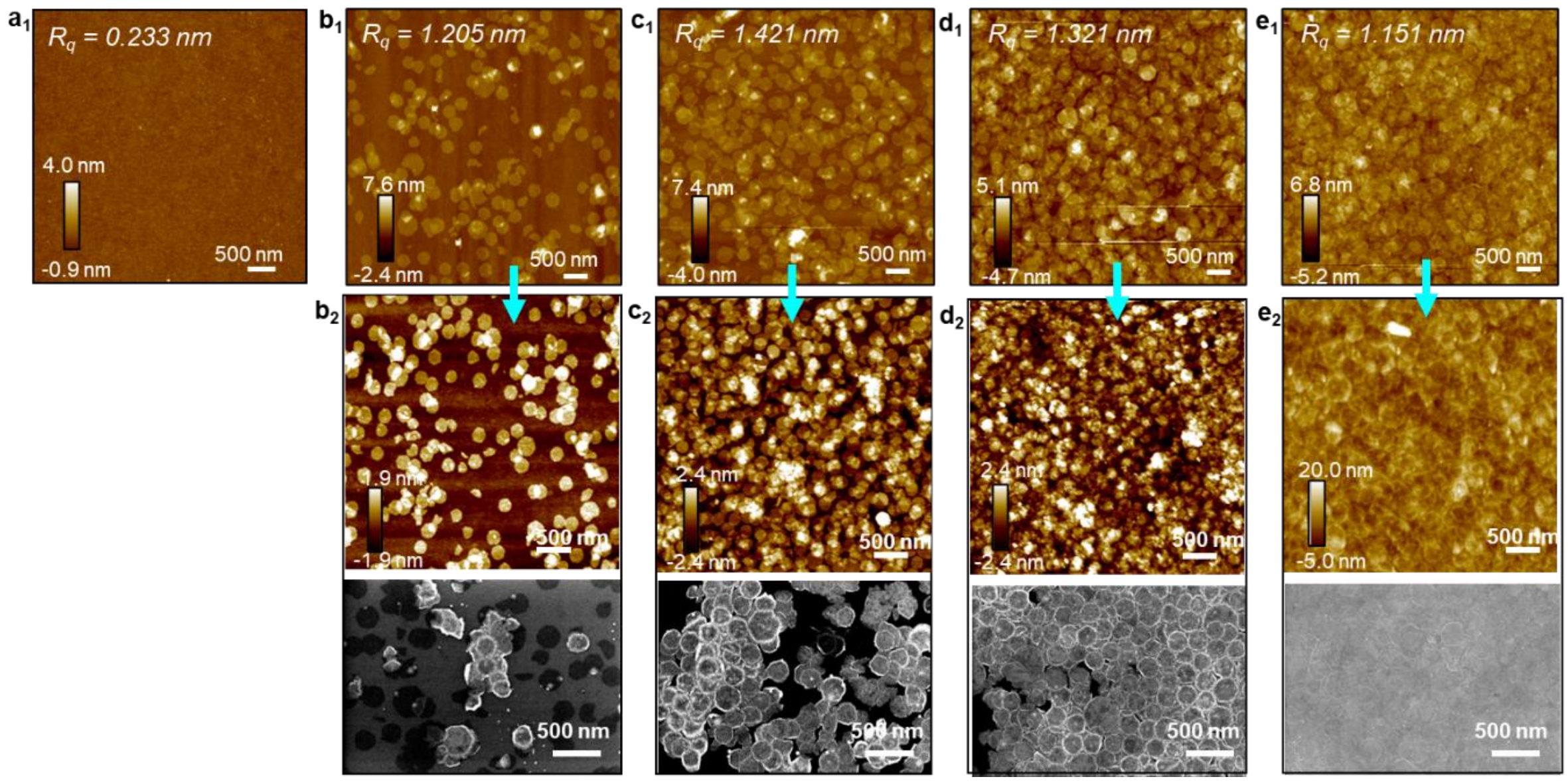

Figure S2. (a$_1$-e$_1$) Two-dimensional (2D) AFM topography for bare *Si* surface (a$_1$) and f-LDH/*Si* samples prepared from different concentrations of SL-LDH solutions: (b$_1$) 2 mg mL^{-1}, (c$_1$) 5 mg mL^{-1}, (d$_1$) 10 mg mL^{-1}, and (e$_1$) 20 mg mL^{-1}. The roughness factors for each f-LDH/*Si* surfaces are calculated over a 5 μm × 5 μm area and are indicated in the respective figure. (b$_2$-e$_2$) AFM for 2D topographic view (upper panel) and SEM images (lower panel) of *den-2DPt$_{waf}$*/f-LDH/*Si* starting from f-LDH/*Si* platforms prepared with different concentrations of SL-LDH solutions: (b$_2$) 2 mg mL^{-1}, (c$_2$) 5 mg mL^{-1}, (d$_2$) 10 mg mL^{-1}, and (e$_2$) 20 mg mL^{-1}.

Among the f-LDH/*Si* samples, the film prepared from a 20 mg mL^{-1} SL-LDH dispersion was found to be optimal, achieving complete wafer-scale surface coverage with minimal surface irregularities, as evidenced by the lowest root-mean-square roughness (R_q) [Figure S2(e$_1$)]. In contrast, LDH-modified *Si* surfaces prepared from lower-concentration SL-LDH dispersions exhibited incomplete surface coverage and non-uniform LDH distribution. Consequently, the optimized f-LDH/*Si* substrate enabled continuous surface-confined Pt growth, resulting in the formation of a wafer-scale *den-2DPt$_{waf}$* film that uniformly covered the entire substrate surface [Figure S2(e$_2$)].

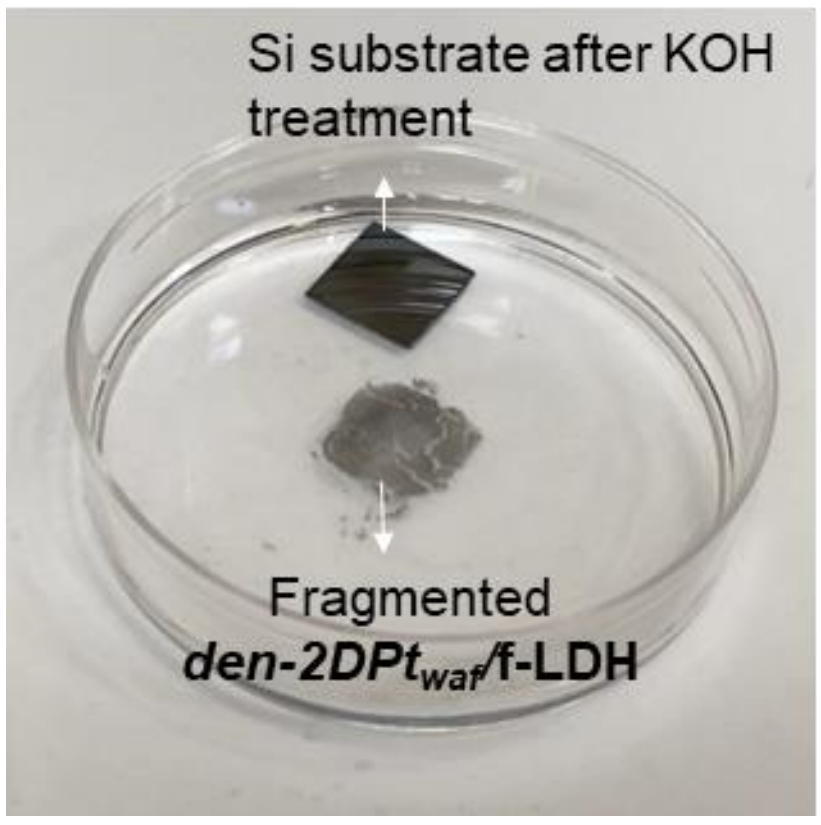

Figure S3. Digital image presenting detachment of *den-2DPt*$_{waf}$/f-LDH from wafer as sacrificial SiO_x layer in *den-2DPt*$_{waf}$/f-LDH/*Si* is removed through aqueous KOH treatment. Note that *Si* represents the starting wafer having SiO_x layer on Si substrate. As evident from the images, the structure completely collapsed into many fragments. Despite this, these fragments provided sufficient morphological information via TEM.

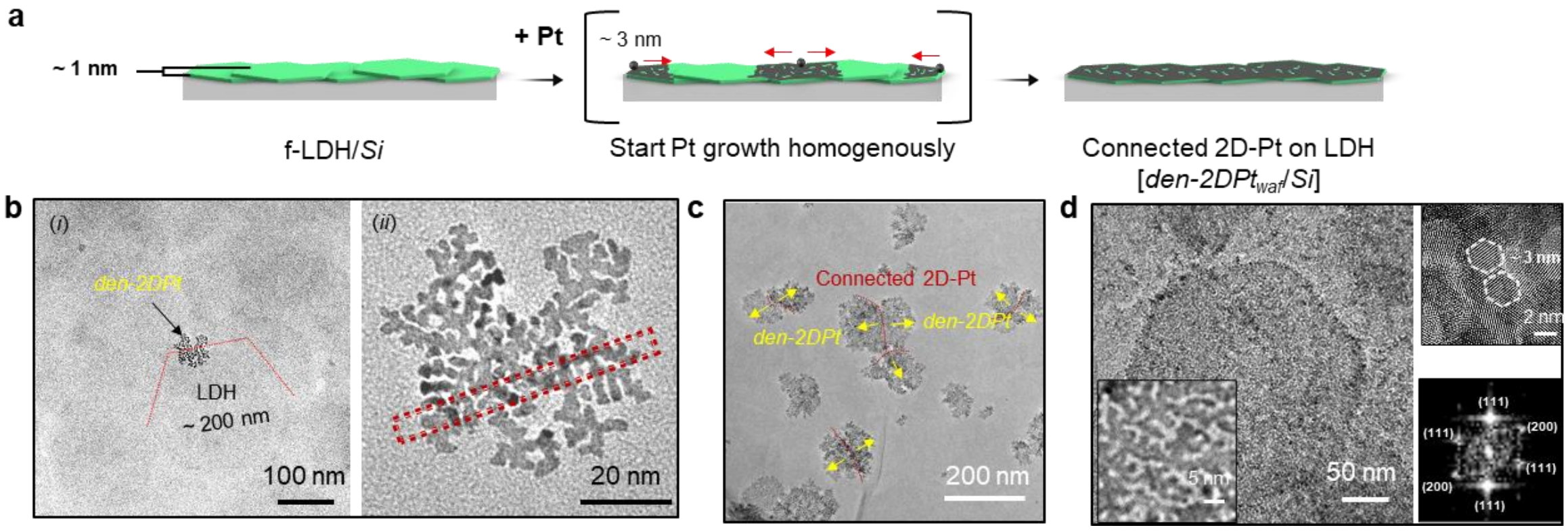

Figure S4. (a) Schematic illustration of the mechanistic growth process of *den-2DPt*$_{waf}$ on a near-flat f-LDH/*Si* surface (R_q = 1.151 nm), initiated by 3-nm-sized Pt growth units through surface-confined growth. The edges of the LDH plate are highlighted with red dotted lines. (b) TEM and HRTEM analyses showing the initial *den-2DPt* growth at the boundaries of LDH edges. (c, d) TEM and HRTEM images of *den-2DPt*$_{waf}$ at intermediate and final growth stages, respectively.

The near-flat surface is critical for uninterrupted surface-confined Pt growth, as the 3-nm Pt crystal size exceeds the height difference between adjacent LDH surfaces (equivalent to the R_q value of 1.15 nm), enabling continuous Pt growth across LDH boundaries.

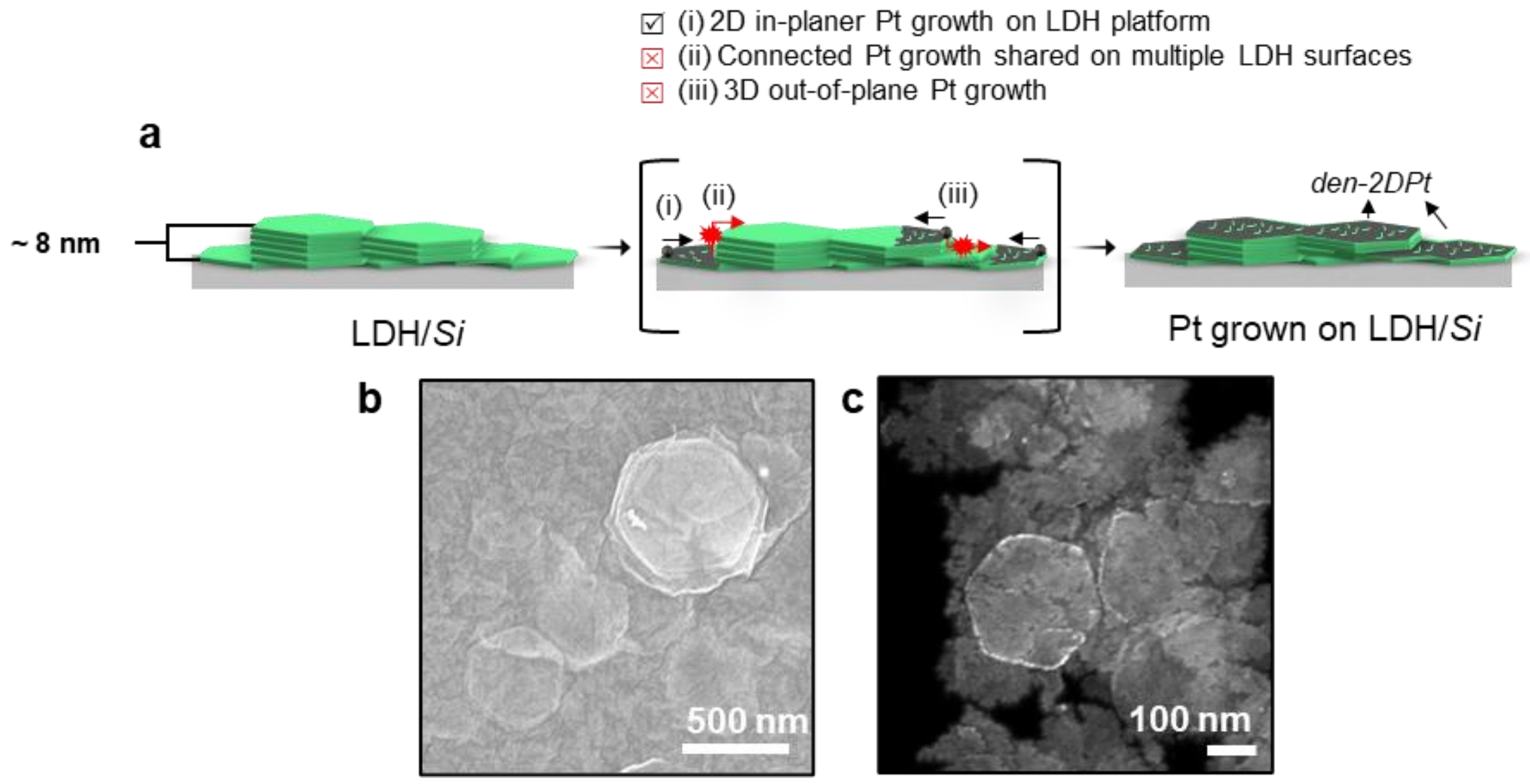

Figure S5. (a) Schematic illustration of Pt growth on roughened LDH/*Si* (R_q = 8.0 nm) under surface-confined growth process. (b) SEM image of LDH/*Si*, prepared by additional drop-casting of SL-LDH (0.04 mL; 50.0 mg mL^{-1}) onto f-LDH/*Si*, followed by air drying without spin-coating. (c) SEM analyses of the Pt product grown on LDH/*Si*, showing a high degree of isolation among individual *den-2DPt* structures formed on separate LDH surfaces.

As anticipated, Pt crystals (~3 nm) were unable to bridge the average height difference between neighboring LDH surfaces i.e., ~8.0 nm, resulting in isolated 2D-Pt structures confined to individual LDH platforms rather than interconnected 2D-Pt sheets. Consequently, continuous, wafer-scale dendritic 2D-Pt growth was not achieved.

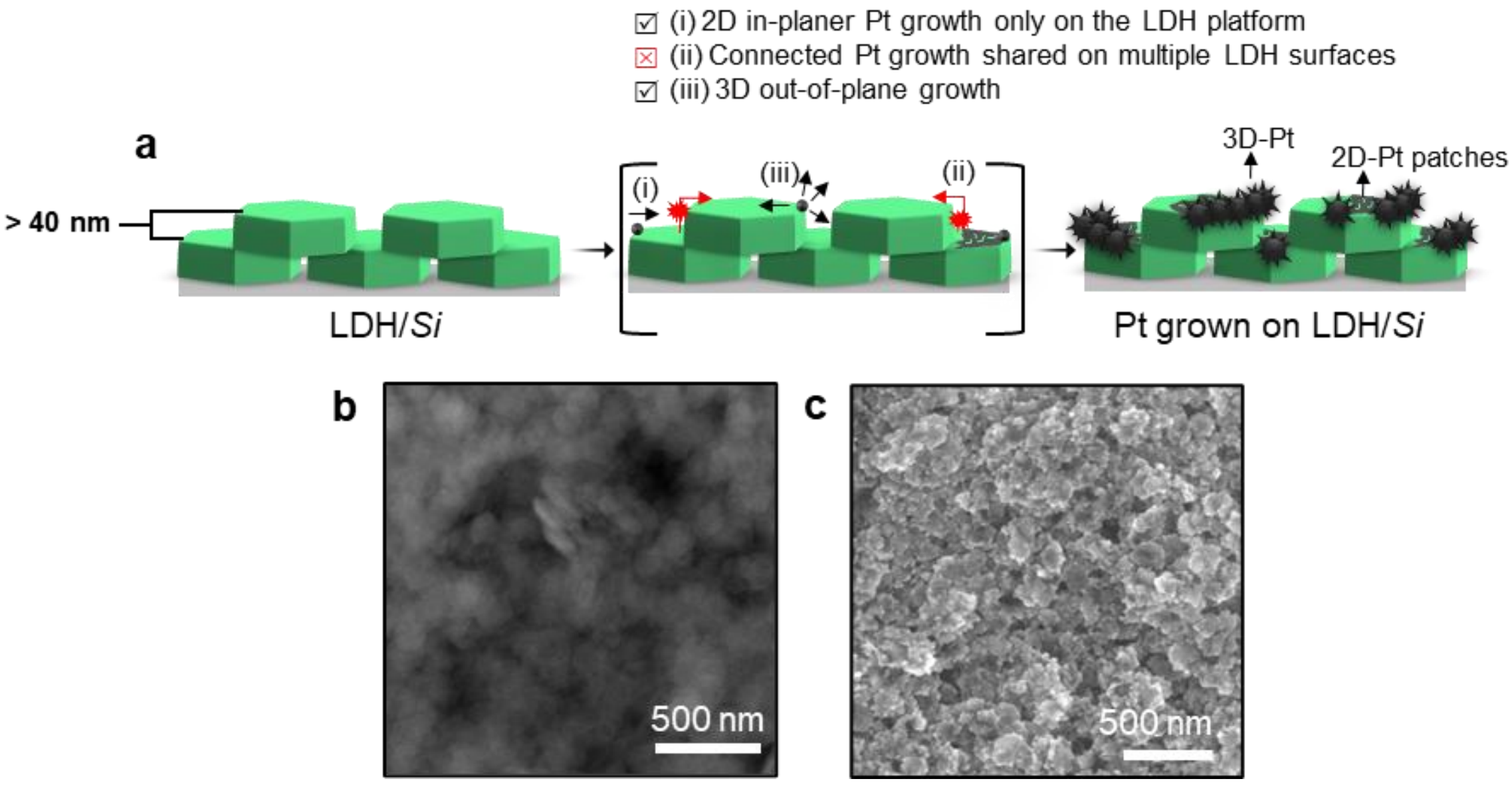

Figure S6. (a) Scheme illustrating the Pt product formed on highly roughened LDH/*Si* substrate (R_q = 48.0 nm) through surface-confined growth. (b) SEM image of the corresponding LDH/*Si* wafer. This roughened substrate surface was prepared using a similar strategy as for f-LDH/*Si*, but with freshly prepared few-layered *(*FL*)*-NiCo LDH (in ethanol, 0.01 mL; 40.0 mg mL^{-1}) following reported protocol with some modification,[S2] spin-coated at 6000 rpm for 60 s. (c) SEM image of the Pt product grown on LDH/*Si*, showing predominantly 3D-Pt growth at the LDH peripheries without fully covering the surface.

Compared to SL-LDH [thickness (*t*) ≈ 1.0 nm] based roughened LDH/*Si* substrate, FL-LDH [*t* ≈ 7.0 nm] based roughened LDH/*Si* is expected to provide larger step-barrier for smooth and continuous Pt growth shared on multiple LDH surfaces. Additionally, the larger edge area of each FL-LDH enhances edge-site reactivity, leading to uncontrollable 3D-Pt growth at the edges rather than propagation along the smooth basal surface.

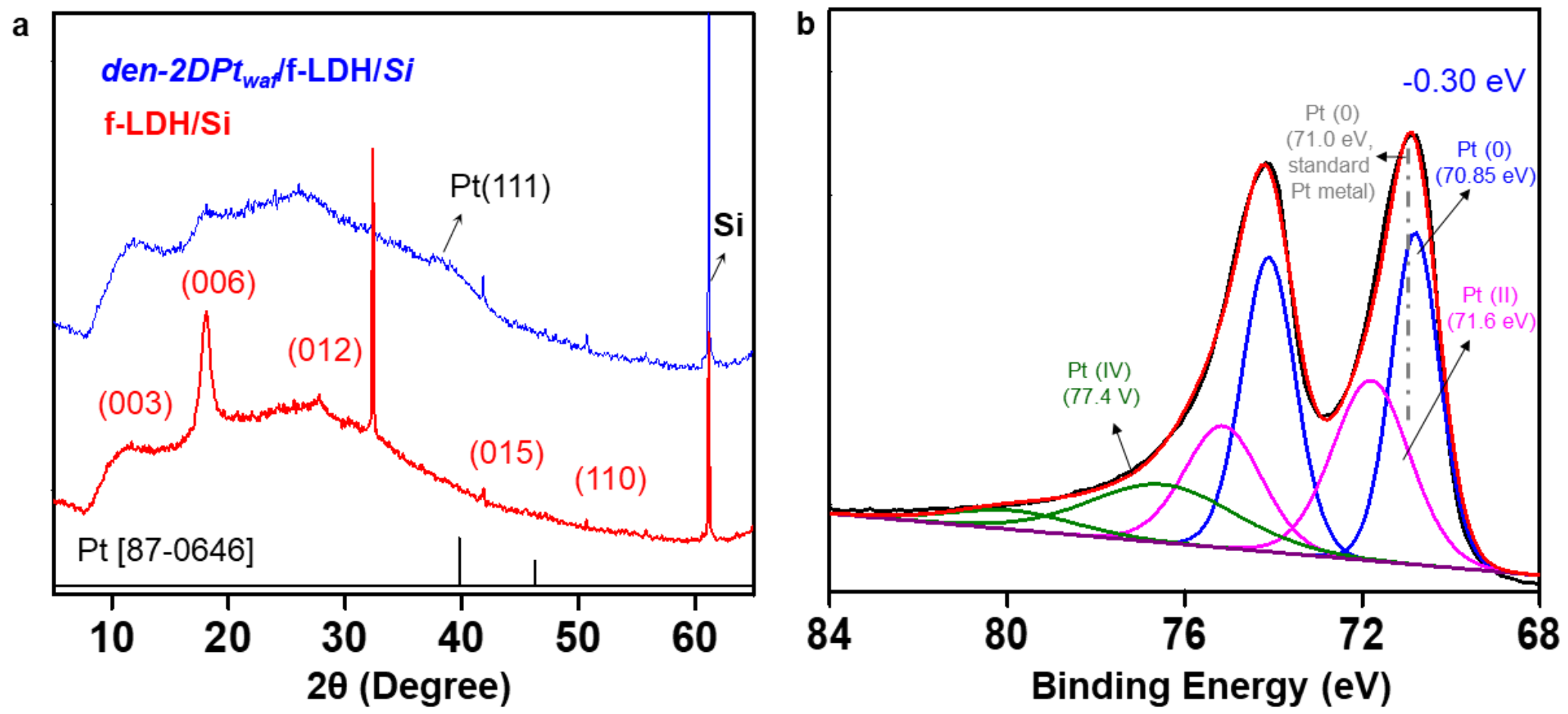

Figure S7. (a) XRD pattern for f-LDH/*Si* (R_q = 1.151 nm) and *den-2DPt$_{waf}$*/f-LDH/*Si*, (b) Pt 4f XPS spectra for isolated *den-2DPt$_{waf}$*/f-LDH highlighting the presence of metallic Pt(0) in the hybrid substrate (mentioned peak position are for Pt $4f_{7/2}$).

On analyzing the XRD of *den-2DPt$_{waf}$*/f-LDH/*Si*, the corresponding peaks for f-LDH have mostly appeared, while the Pt's nanocrystal pattern is almost invisible, probably owing to their few-nm-thin thickness present on f-LDH/*Si*. However, XPS analyses verify the presence of metallic Pt^0 at 70.7 eV (for Pt $4f_{7/2}$) in significant quantity in *den-2DPt$_{waf}$*/f-LDH alongside the unavoidable presence of PtO_x [Pt $4f_{7/2}$ at 71.8 and 76.7 eV for Pt(II) and Pt(IV), respectively] at the surface. Notably, the probable Pt/f-LDH electronic interactions have caused peak shifting by at least -0.30 eV than the standard Pt-metal (Pt $4f_{7/2}$ at 71.0 eV).[S1]

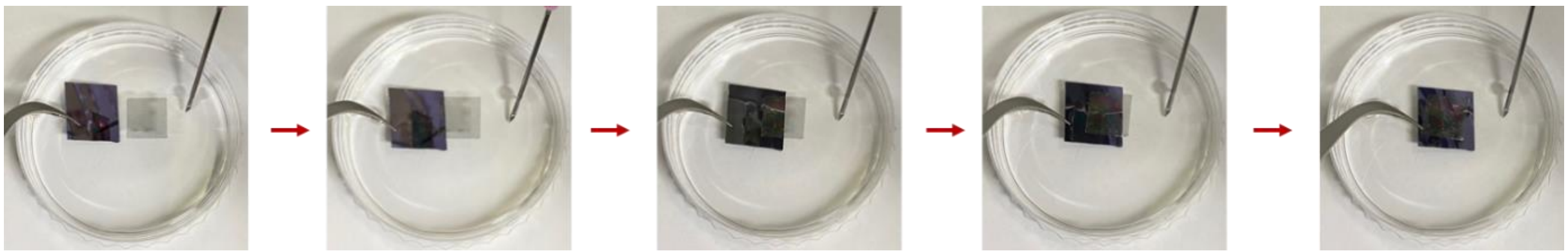

Figure S8. Snapshots at different steps followed for smooth transferring of detached [PMMA] *den-2DPt*$_{waf}$ on fresh *Si* substrate.

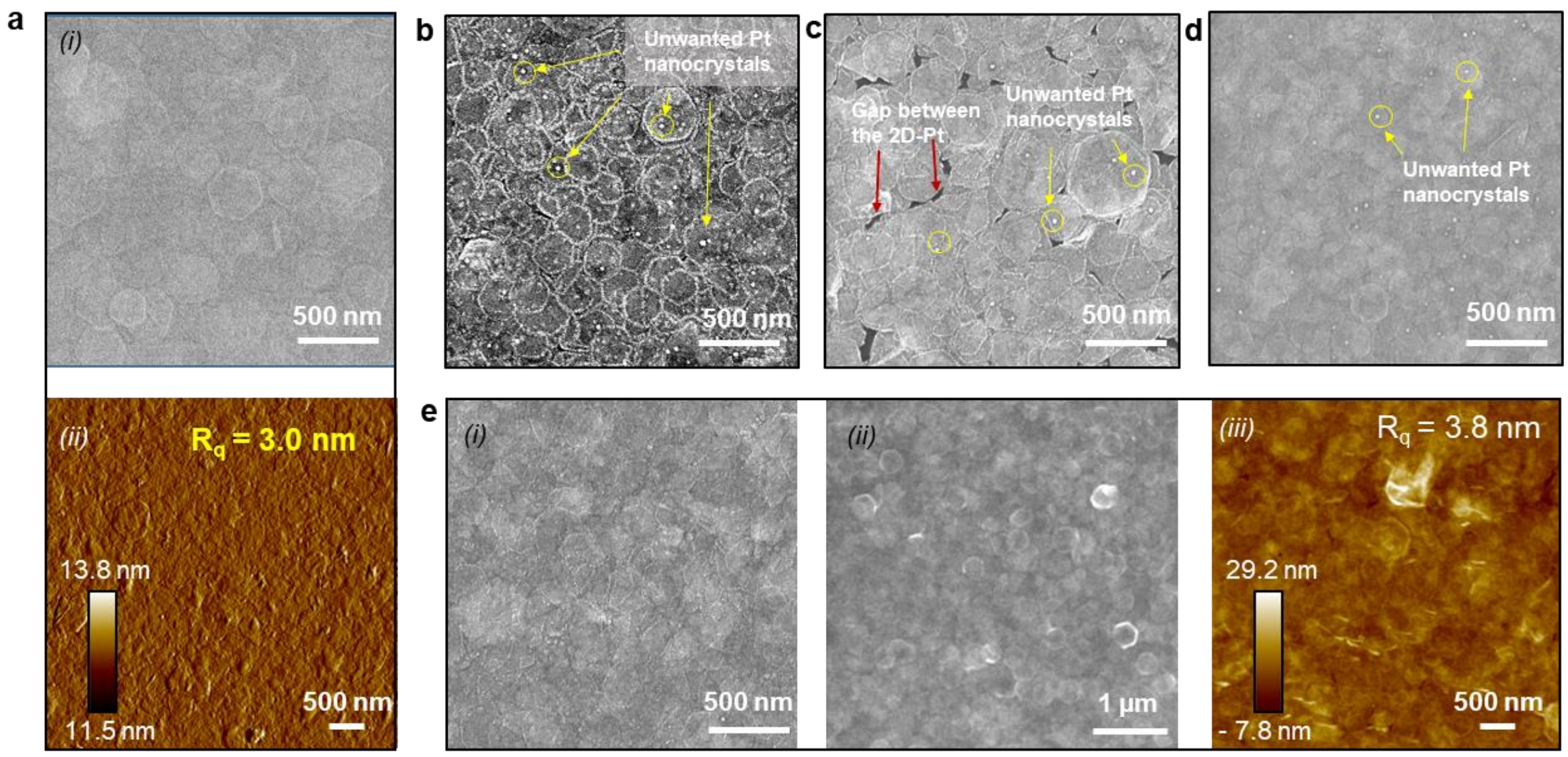

Figure S9. (a) SEM (*i*) and AFM (*ii*) images f-LDH on [3 cm x 3 cm] sized *Si* wafer. SEM image of the synthesized 2D-Pt on f-LDH/*Si* in presence of various amount of PVP: (b) 10 mg, (c) 20 mg, (d) 30 mg, (e) 45 mg to optimize the uniform growth of 2D-Pt covering the f-LDH/*Si* and minimize unrestricted Pt nanocrystal synthesis.

The highlighted Pt nanocrystals correspond to nanoparticles formed via bulk-solution reduction of Pt precursors rather than LDH-confined growth. Such particles are undesirable because they compete with template-directed 2D Pt formation and reduce film uniformity. PVP suppresses this bulk nucleation process and promotes selective Pt growth on the LDH surface.

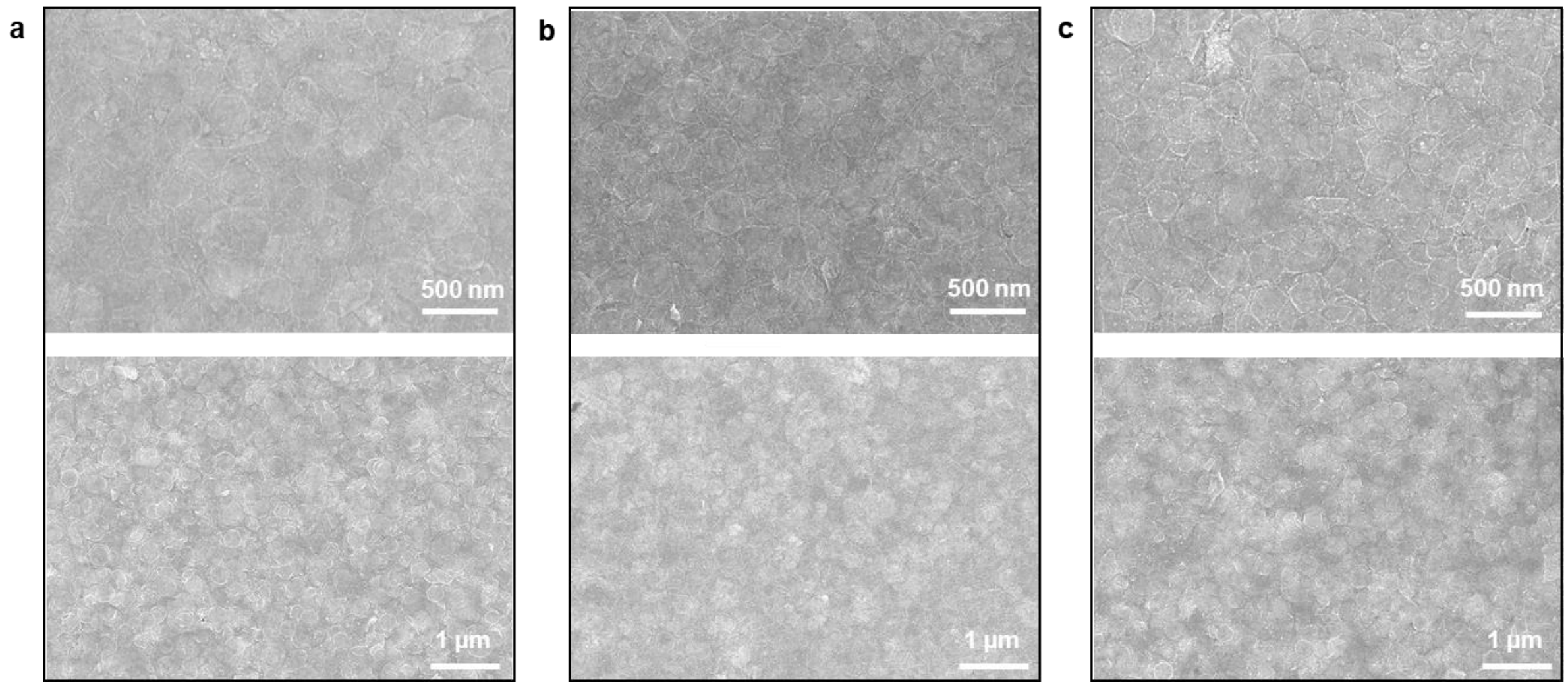

Figure S10. SEM image of the synthesized 2D-Pt on [5 cm x 5 cm] sized f-LDH/*Si* in presence of various amount of PVP (a) 45 mg, (b) 50 mg, (c) 55 mg.

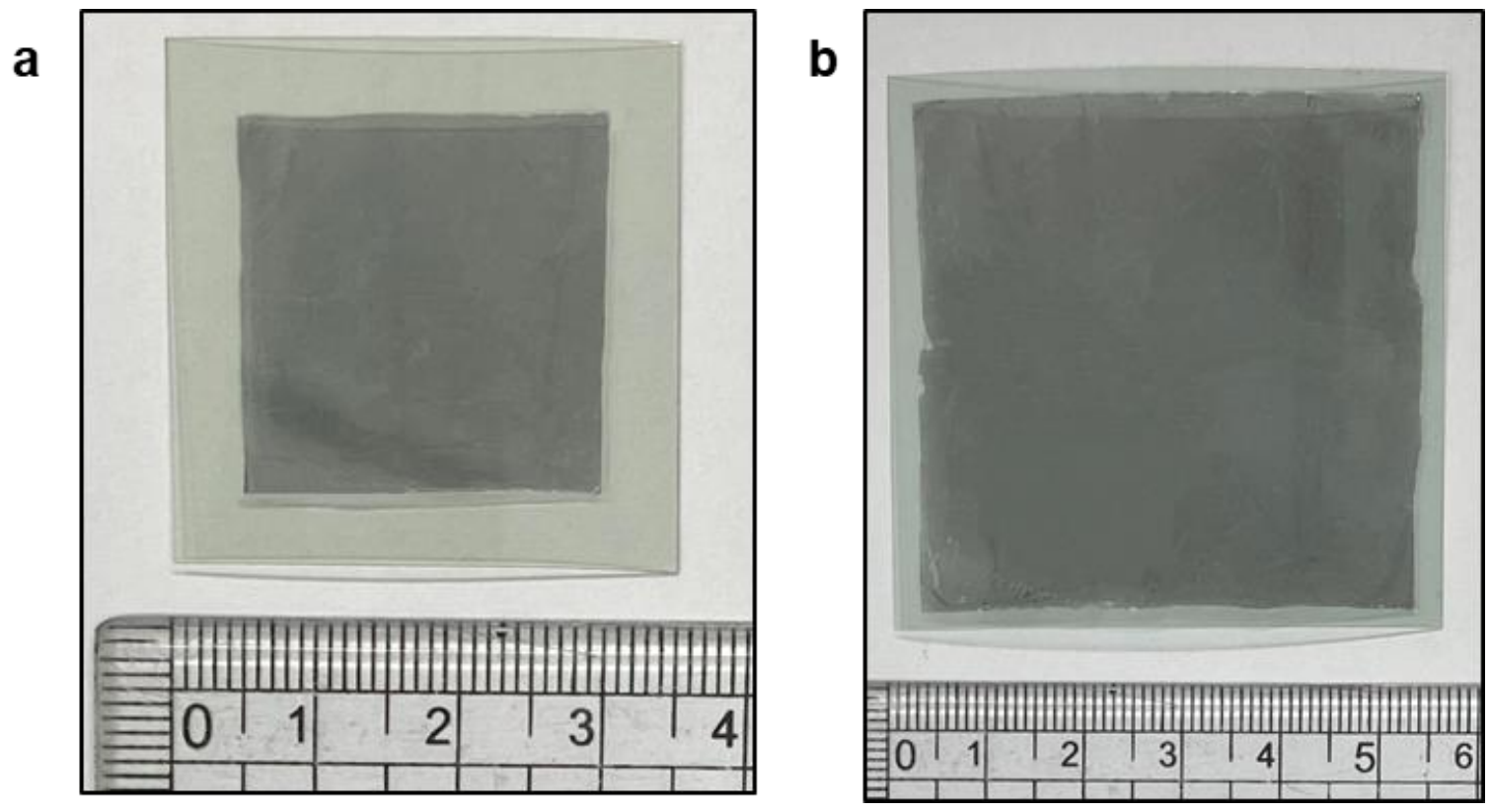

Figure S11. Digital images of transferred *den-2DPt$_{waf}$* of different dimensions on fresh PET substrate: (a) 3 cm × 3 cm and (b) (a) 5 cm × 5 cm.

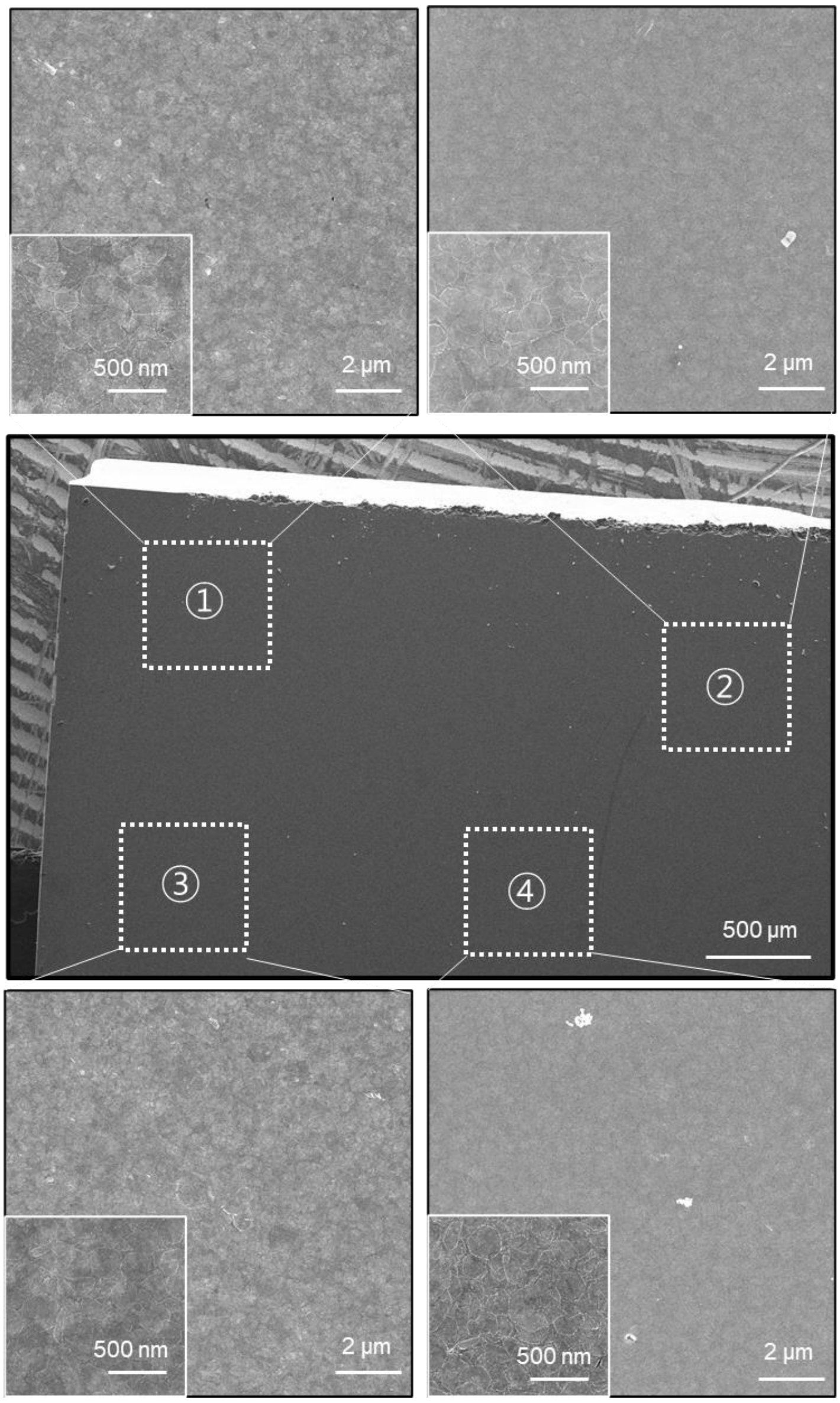

Figure S12. SEM images of transferred *den-2DPt$_{waf}$* on fresh Si substrate demonstrate a damage-free transfer process with uniform large-area coverage and excellent retention of the Pt nanoarchitecture.

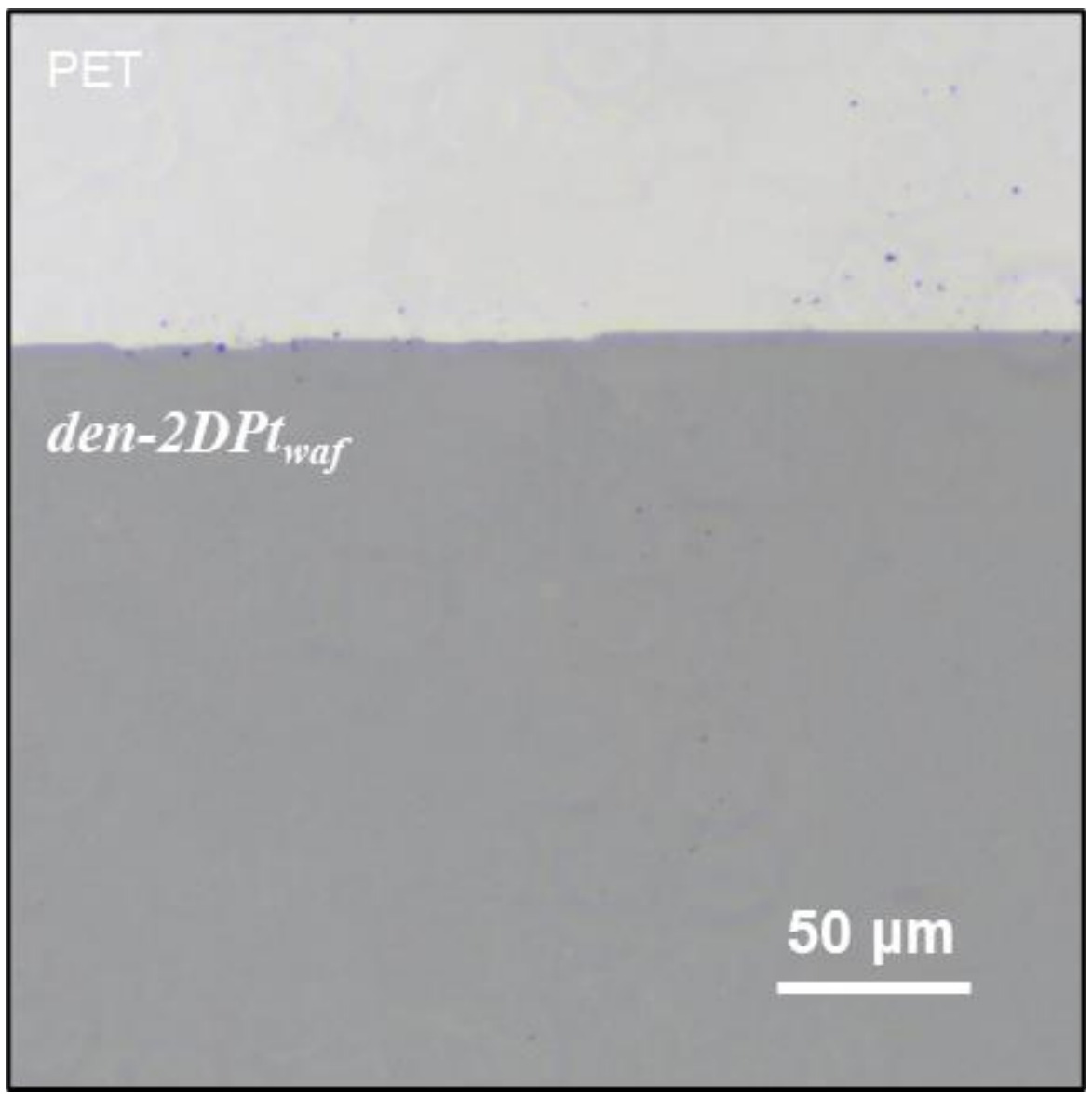

Figure S13. Optical microscopy image of transferred *den-2DPt*$_{waf}$ on fresh PET substrate, taken under a white light.

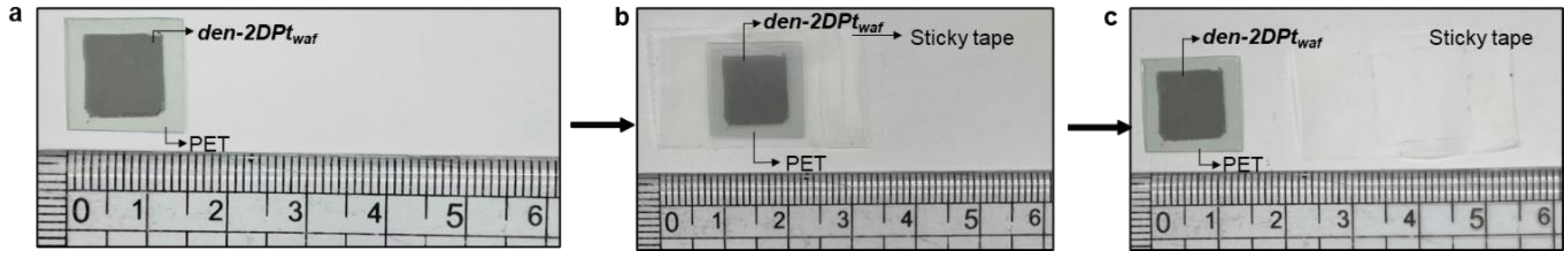

Figure S14. Images showing good adhesion between transferred *den-2DPt*$_{waf}$ on PET substrate: (a) the nanosheet film on PET, (b) after taping a sticky tape, and (c) after peeling off the sticky tape.

As clear in Figure S10c, Pt parts (blackish imprint) are absent on the white-colored sticky tape after peeling off from the Pt transferred PET surface.

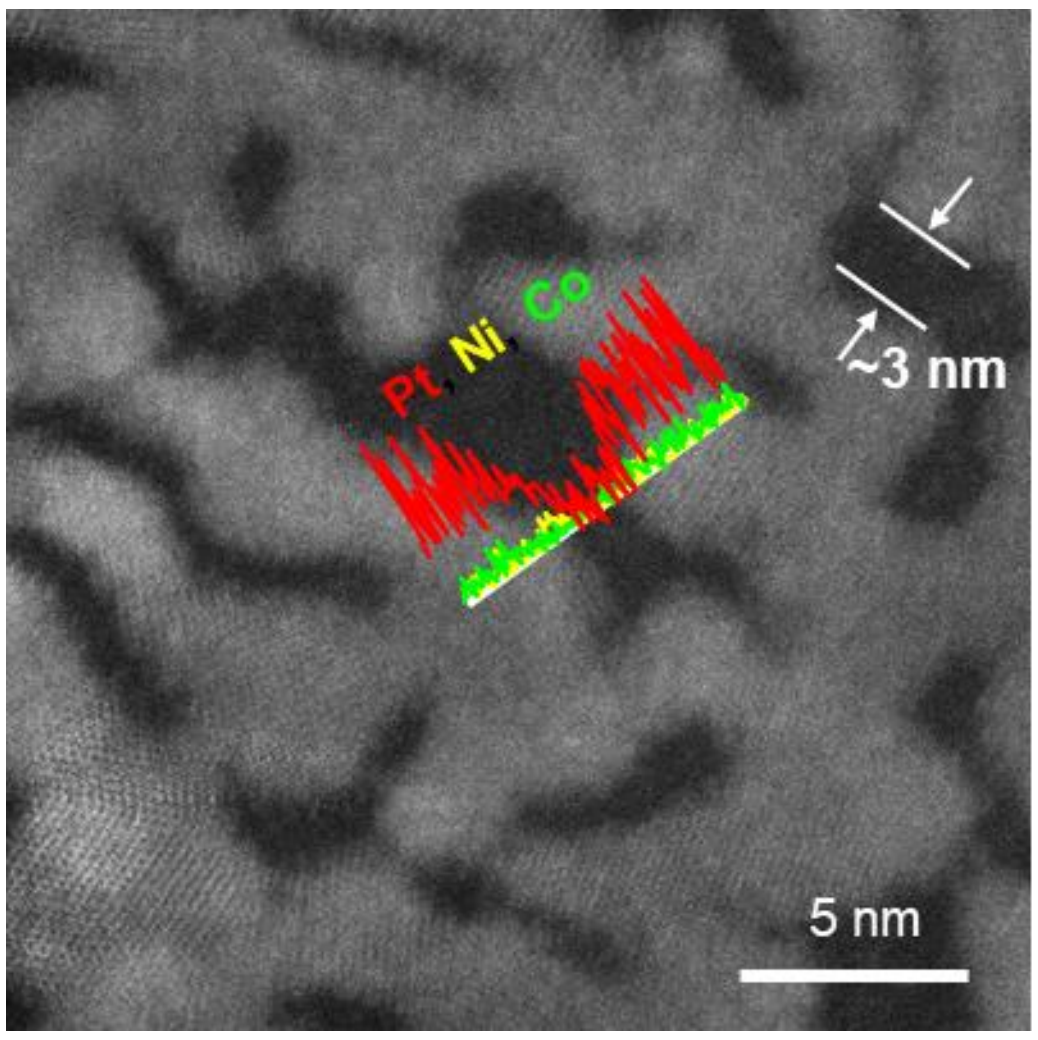

Figure S15. HAADF-STEM image of transferred *den-2DPt*$_{waf}$ highlighting nanoscale gaps between Pt branches. EDS elemental mapping confirms the absence of Ni and Co, indicating complete removal of the LDH template and high purity of the transferred Pt film.

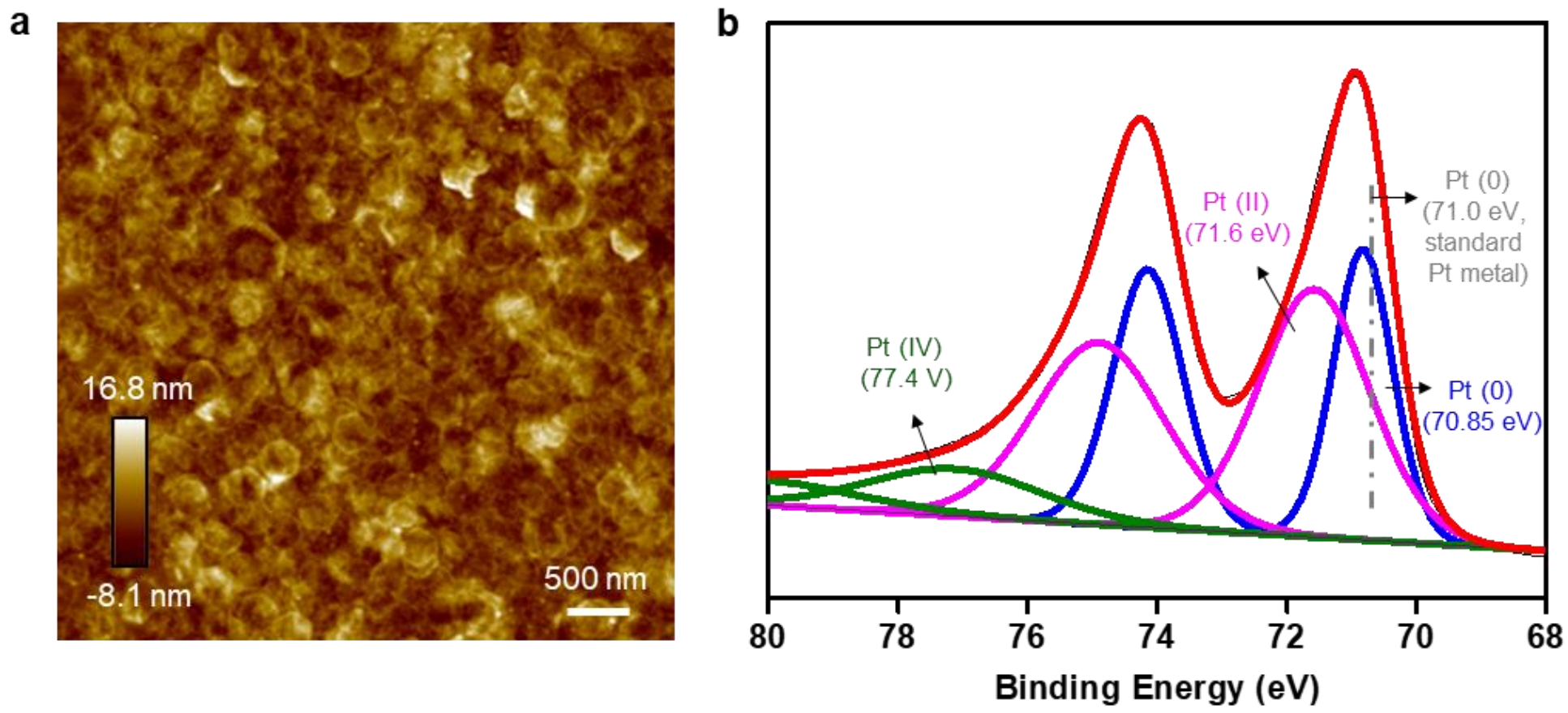

Figure S16. (a) Topographic AFM for 1 cm^2 transferred *den-2DPt*$_{waf}$ on fresh *Si* substrate. (b) Pt 4f XPS spectra for transferred *den-2DPt*$_{waf}$ on *Si* substrate (mentioned peak position are for Pt $4f_{7/2}$).

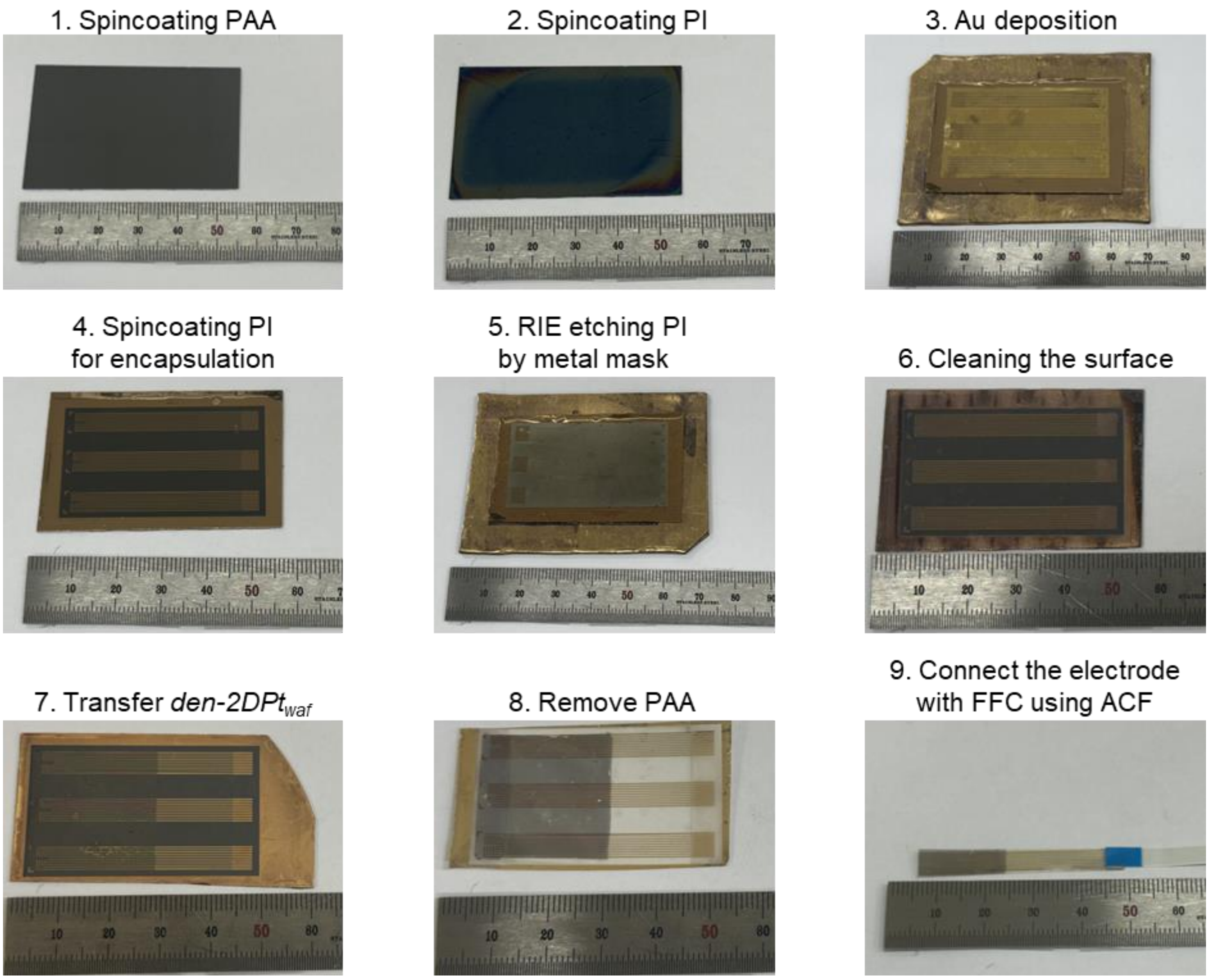

Figure S17. Fabrication procedures of polyimide (PI)/Au substrate coated with transferred *den-2DPt*$_{waf}$.

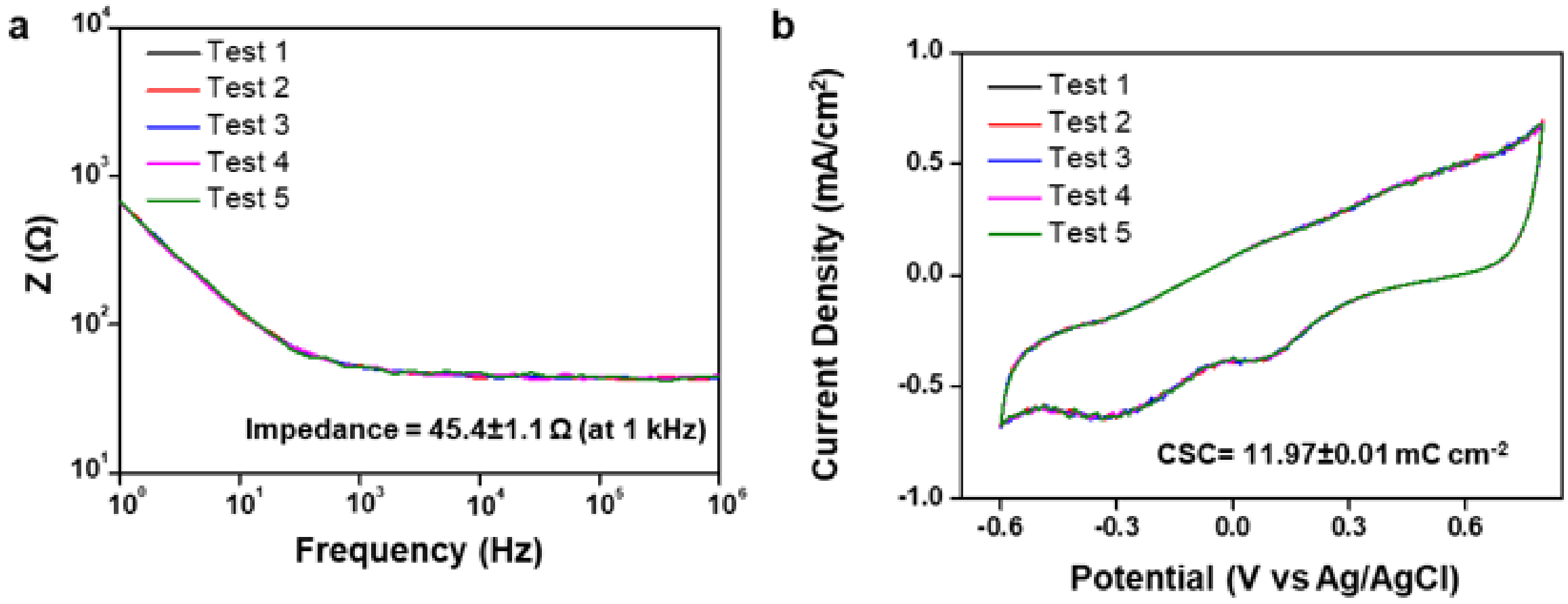

Figure S18. Reproducibility assessment for electrochemical features of *den-2DPt*$_{waf}$/Au electrode. (a) EIS spectra recorded with a 50 mV sinusoidal perturbation and (b) CV profiles measured at a scan rate of 50 mV s^{-1} in Ar-saturated PBS (0.01 M, pH 7.4) for independently fabricated *den-2DPt*$_{waf}$/Au electrodes. The corresponding averaged electrochemical characteristics are presented as mean ± standard deviation (n = 5) in the respective panels.

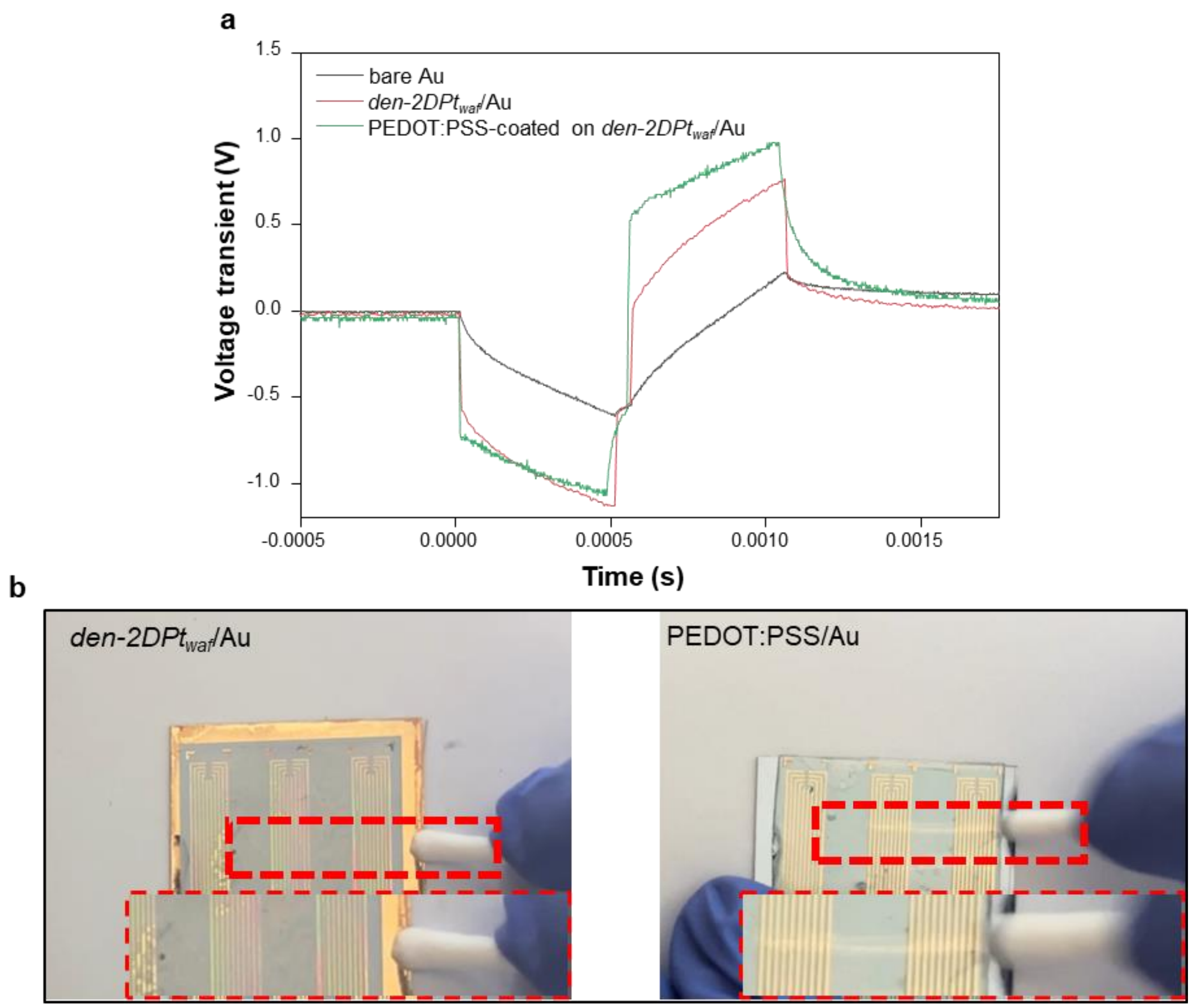

Figure S19. (a) CIC measurements showing the performance enhancement of *den-2Dpt*$_{waf}$/Au after PEDOT:PSS coating. (b) Digital images illustrating the fragile nature of PEDOT:PSS, highlighting its limitation for long-term use.

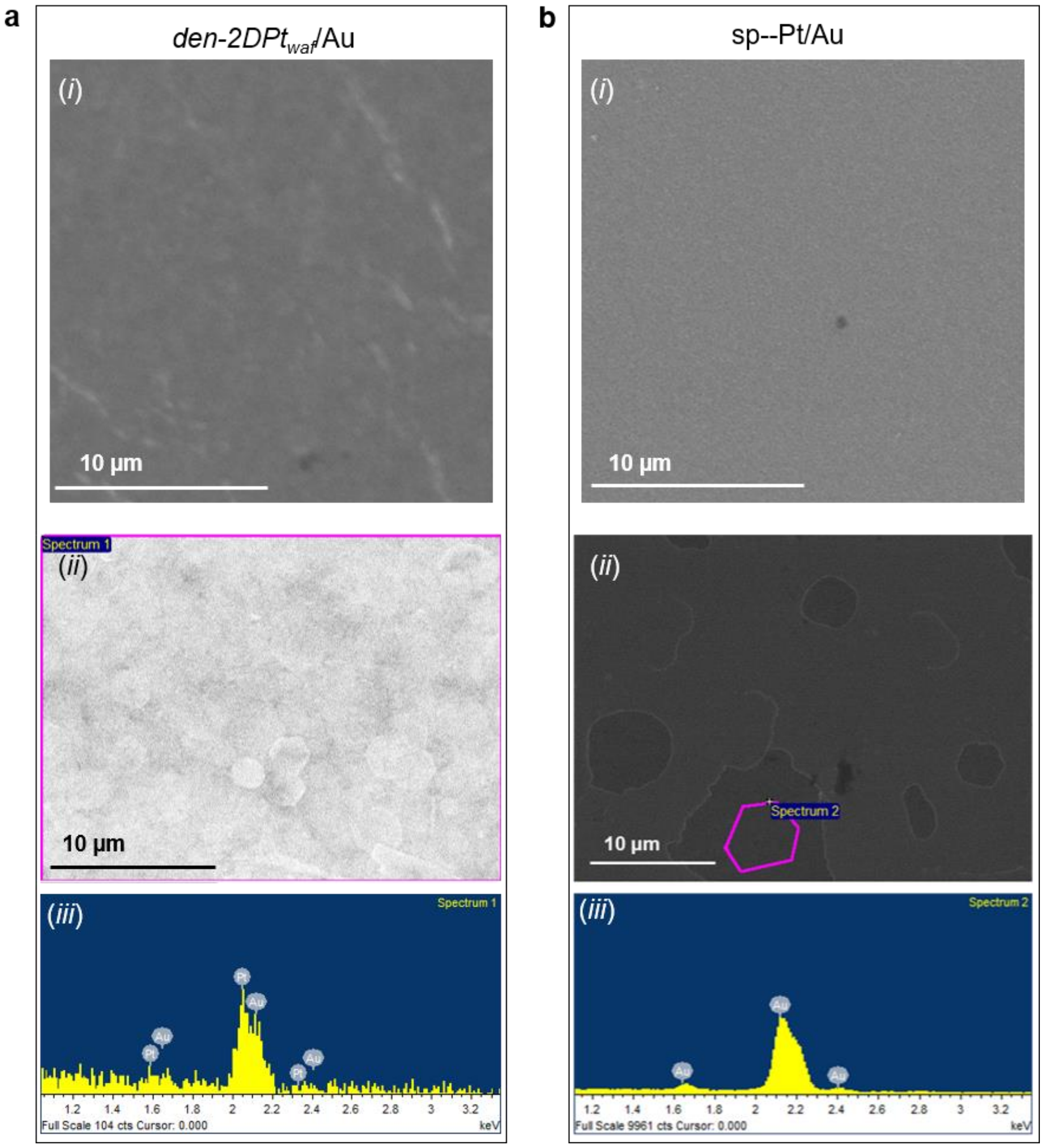

Figure S20. SEM images comparing the morphology of (a) *den-2DPt$_{waf}$*/Au and (b) sp-Pt/Au before (*i*) and after (*ii*,*iii*) the oxidative aging test (PBS + H_2O_2, 85 °C, 7 days). A clear surface alteration and damage are observed for the initially smooth sp-Pt electrode after aging, whereas no delamination, disintegration, or significant morphological changes are observed on the *den-2DPt$_{waf}$* surface. Corresponding EDS analyses in panel (*iii*) reveal pronounced macro-pit formation exclusively on sp-Pt/Au, while the *den-2DPt$_{waf}$*/Au electrode remains structurally unchanged under identical conditions.

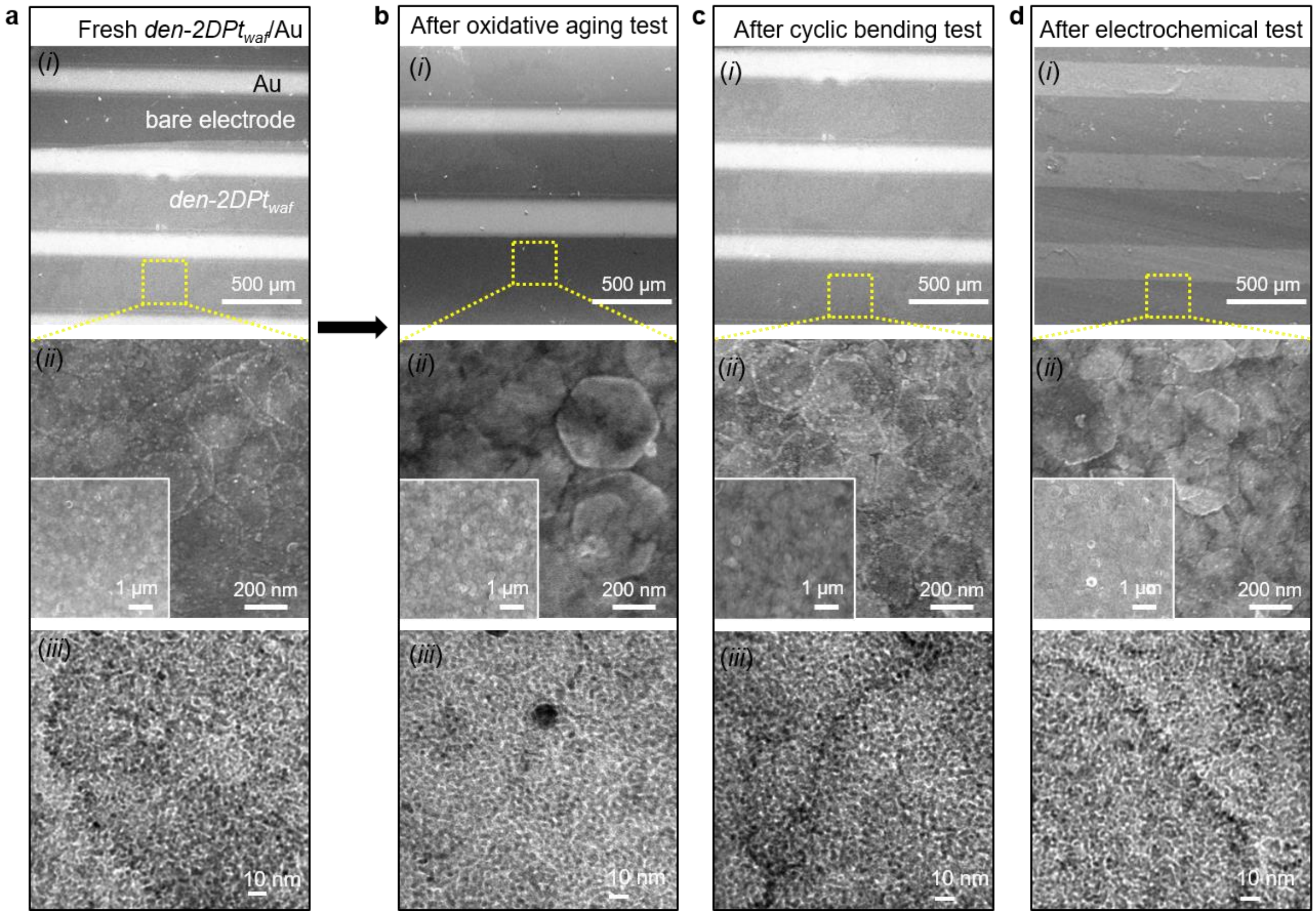

Figure S21. Comparative SEM (*i, ii*) and TEM (*iii*) images of the *den-2DPt$_{waf}$*/Au electrode recorded (a) before testing, (b) after the electrochemical aging test (PBS + H_2O_2, 85 °C, 7 days), (c) after the mechanical flexibility test and (d) after the electrochemical property test showing negligible nanoscale morphological changes. The corresponding higher-magnification SEM images in panel (*ii*) further confirm that the hexagonal Pt disks, with lateral dimensions of several hundred nanometers, remain structurally intact after both tests. TEM images were recorded for determining any potential pore change or disruption in *den-2DPt$_{waf}$*. It is evident that pores remain stable after the chemical or mechanical stress tests.

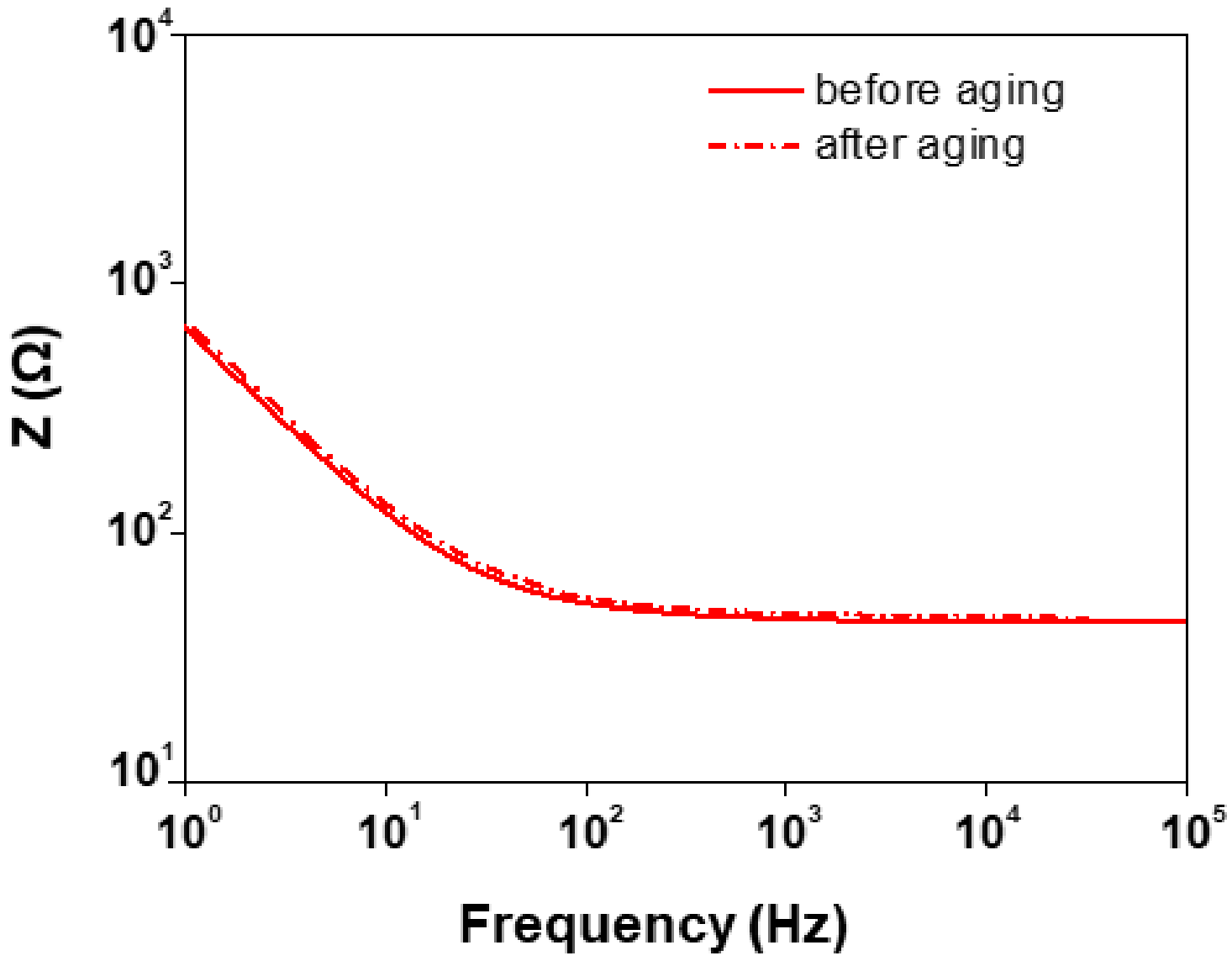

Figure S22. EIS spectra of the *den-2DPt$_{waf}$*/Au electrode recorded before and after the aging test using a 50 mV sinusoidal perturbation, showing comparable impedance responses across the measured frequency range, indicative of a stable electrode interface after aging.

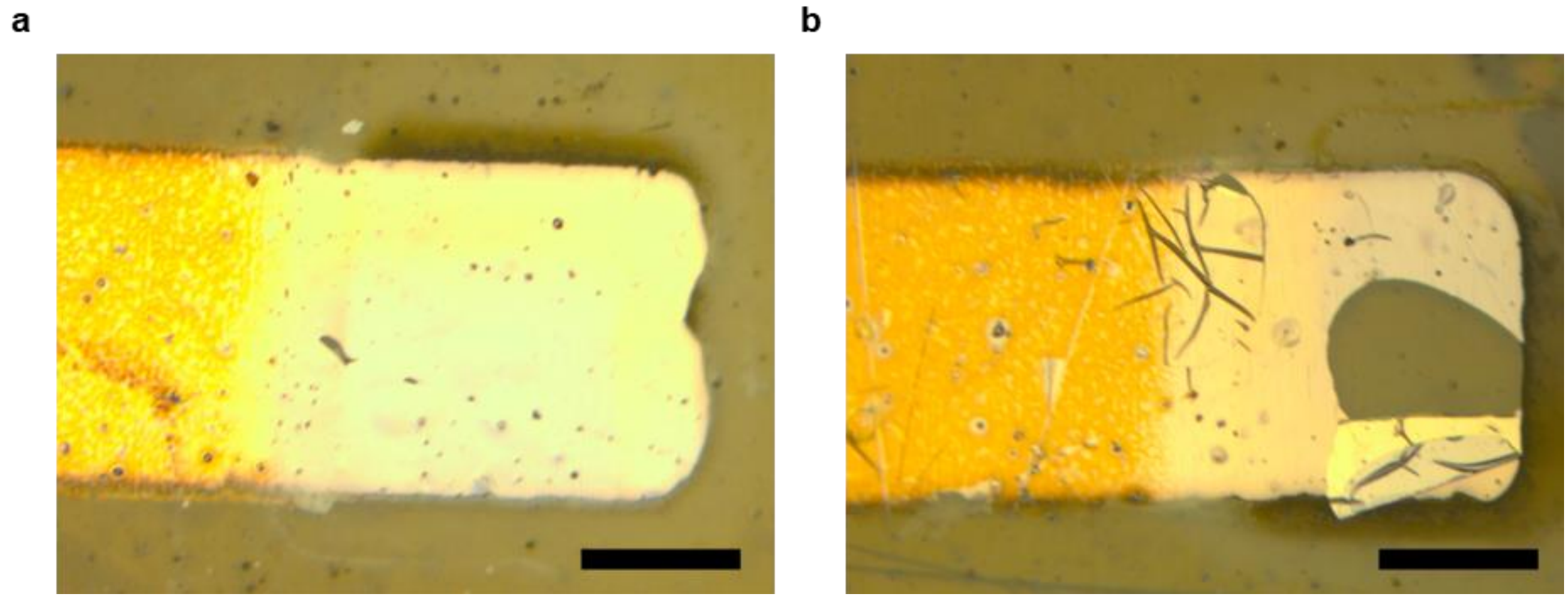

Figure S23. Optical microscopy images of the sp-Pt/Au (a) before and (b) after accelerating aging test. Scale bar: 100 μm.

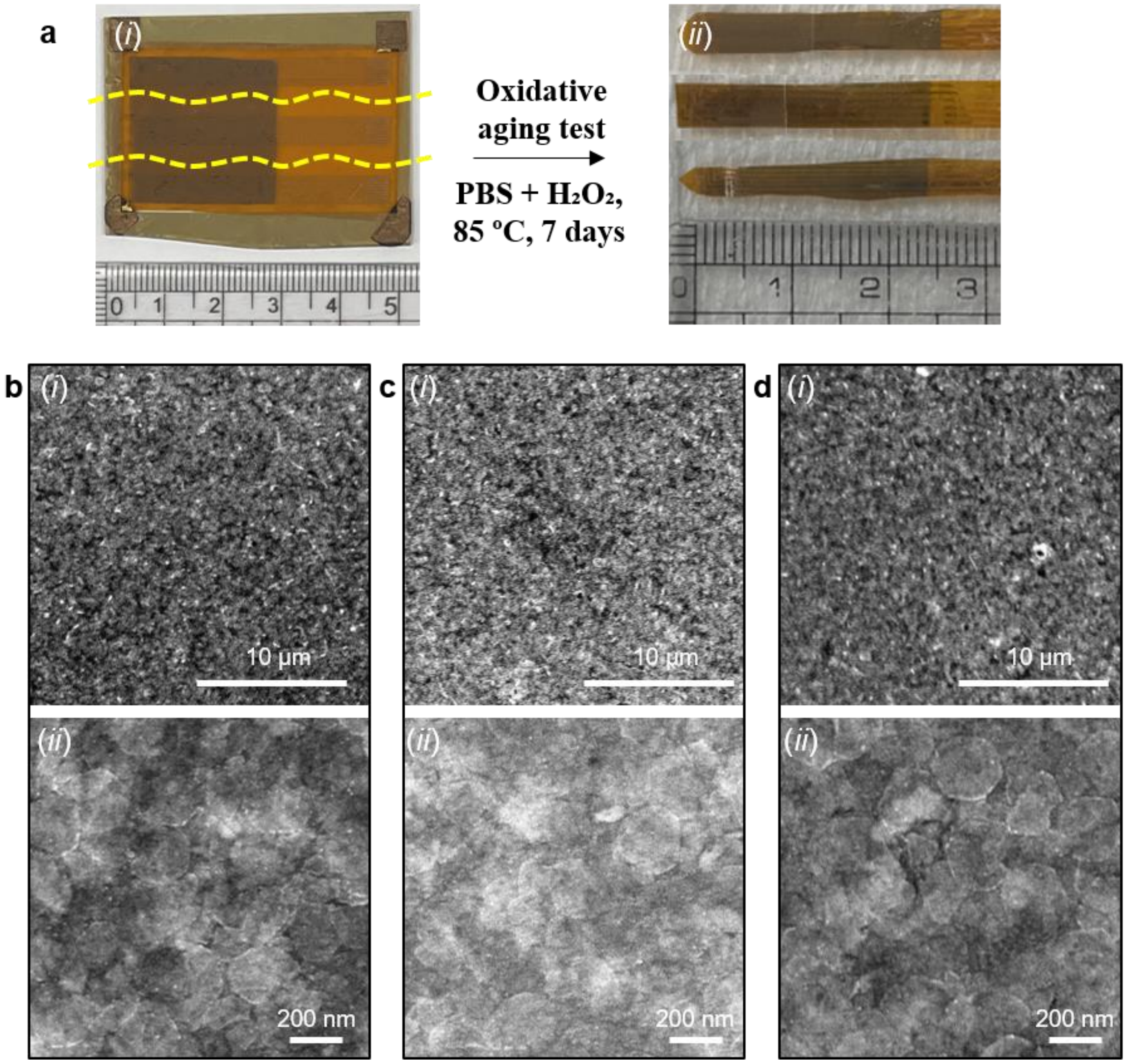

Figure S24: SEM analysis demonstrating the oxidative-stress resistance and reproducibility of *den-2DPtwaf*/Au in PBS containing H_2O_2 at 85 °C for 7 days. (a) Digital images of the as-fabricated den-2DPtwaf/Au sample (i), used to divide the same large-area fabricated electrode into three sections for three independent oxidative-aging tests. Yellow dashed lines indicate the cutting positions used to divide the sample into three sections prior to aging. (ii) Digital images of the three electrodes after oxidative aging. (b–d) Low- (i) and high-magnification (ii) SEM images of the three sections after oxidative aging, corresponding to the top, middle, and bottom regions of the sample shown in (a-ii), respectively.

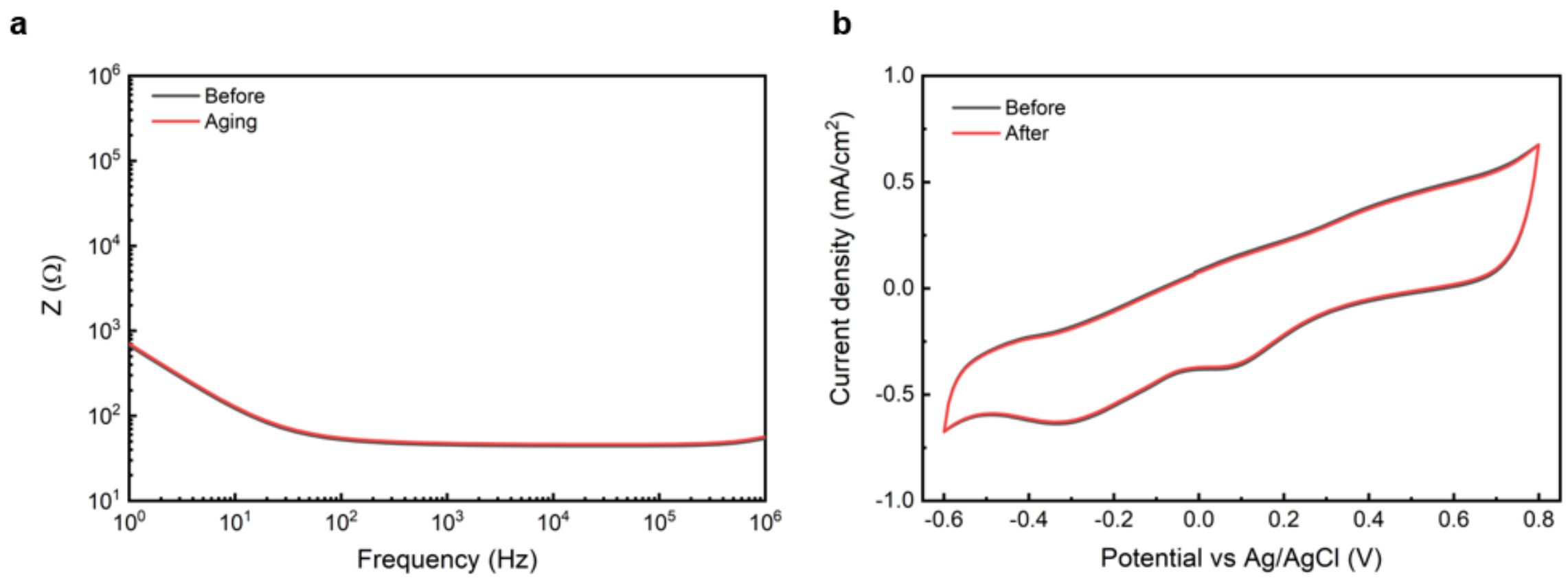

Figure S25. **Long-term electrochemical cycling stability of the *den-2DPt$_{waf}$*/Au electrode after repetitive electrical stimulation.** (a) Electrochemical impedance spectroscopy (EIS) spectra and (b) cyclic voltammetry (CV) curves measured before and after 50,000 biphasic stimulation pulses in a simulated physiological environment. The electrode retained nearly identical electrochemical characteristics after stimulation, with only a 5.66% increase in impedance at 1 kHz and a 4.75% decrease in CSC.

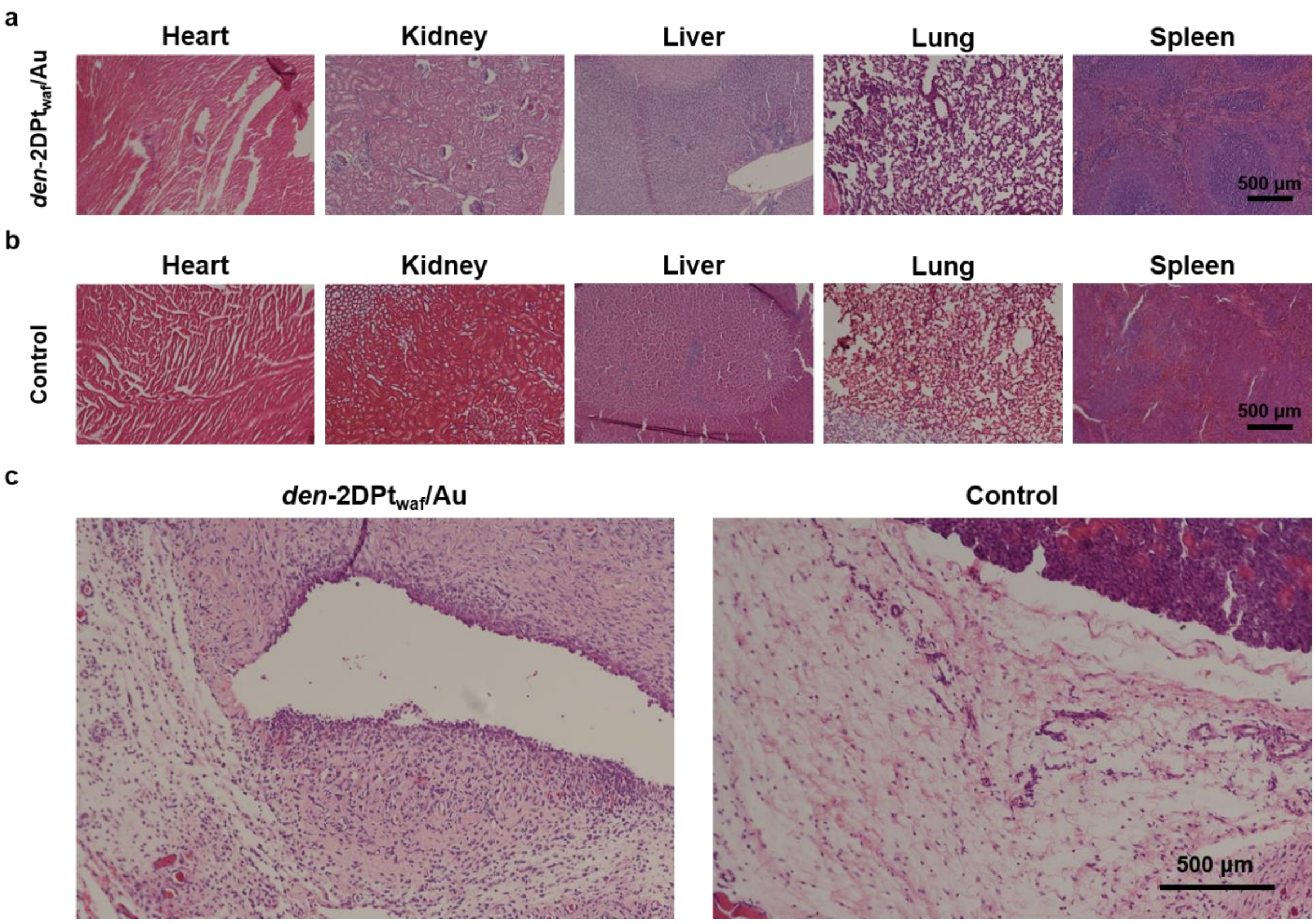

Figure S26. **Qualitative histological assessment 2 weeks after VNS electrode implantation.** Representative hematoxylin and eosin (H&E)-stained sections from one implanted animal and one sham-operated control animal (implant, n = 1; sham, n = 1). (a) Heart, kidney, liver, lung and spleen from the implanted animal. (b) Heart, kidney, liver, lung and spleen from the sham-operated control animal. (c) Local implantation-site tissue from the implanted animal (left) and the corresponding implant-pocket tissue from the sham-operated control animal (right). The sham animal underwent the same surgical incision and formation of the subcutaneous implant pocket without electrode placement, The implantation-site specimen from the implanted animal included the immediate peri-electrode subcutaneous tissue. Images were acquired at 10× objective magnification.

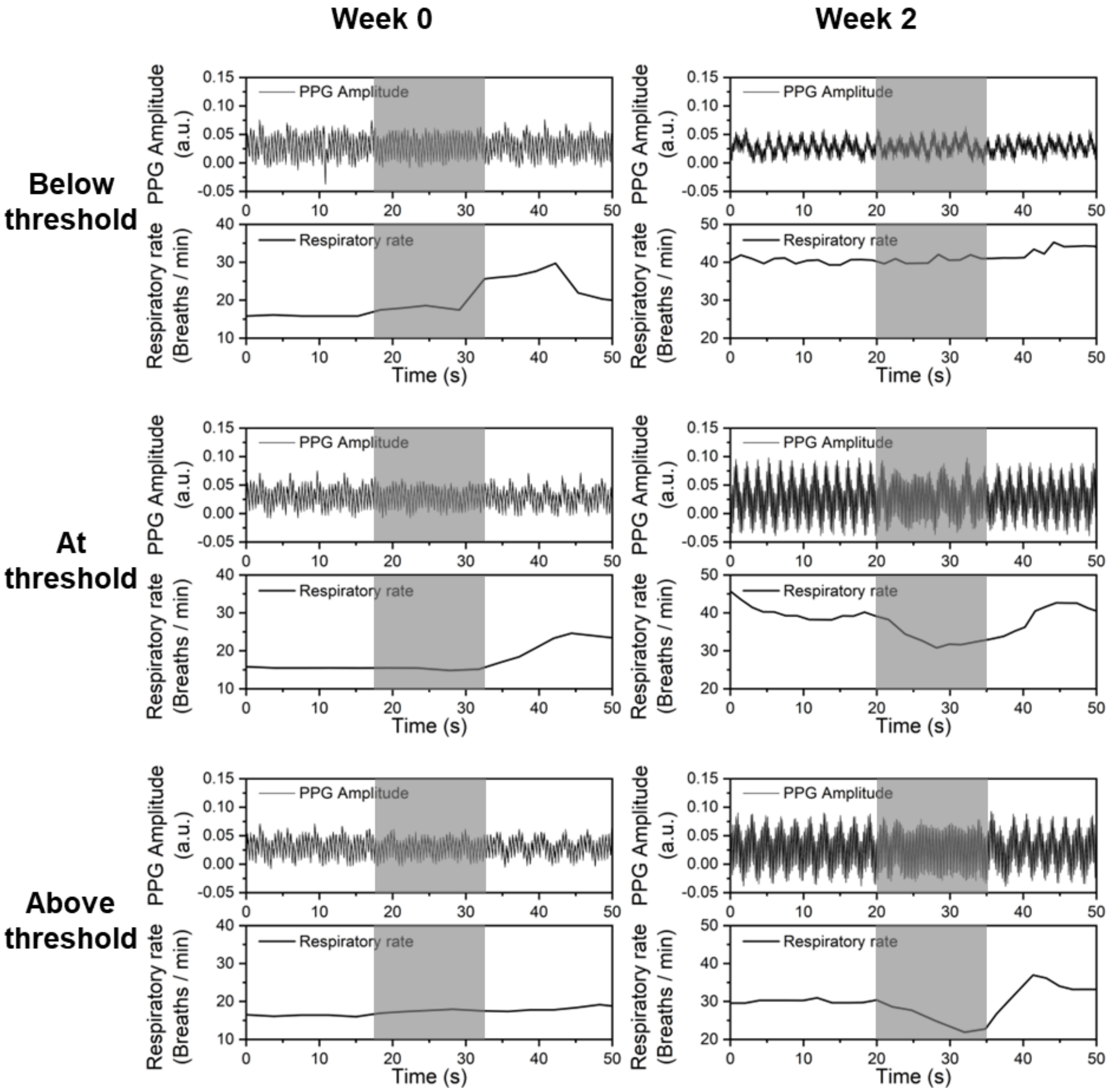

Figure S27. PPG-derived respiratory waveform recorded below (upper), at (middle), and above (lower) the functional stimulation threshold at 0 weeks and 2 weeks post-implantation. The gray region indicates the period during which vagus nerve stimulation (VNS) was applied.

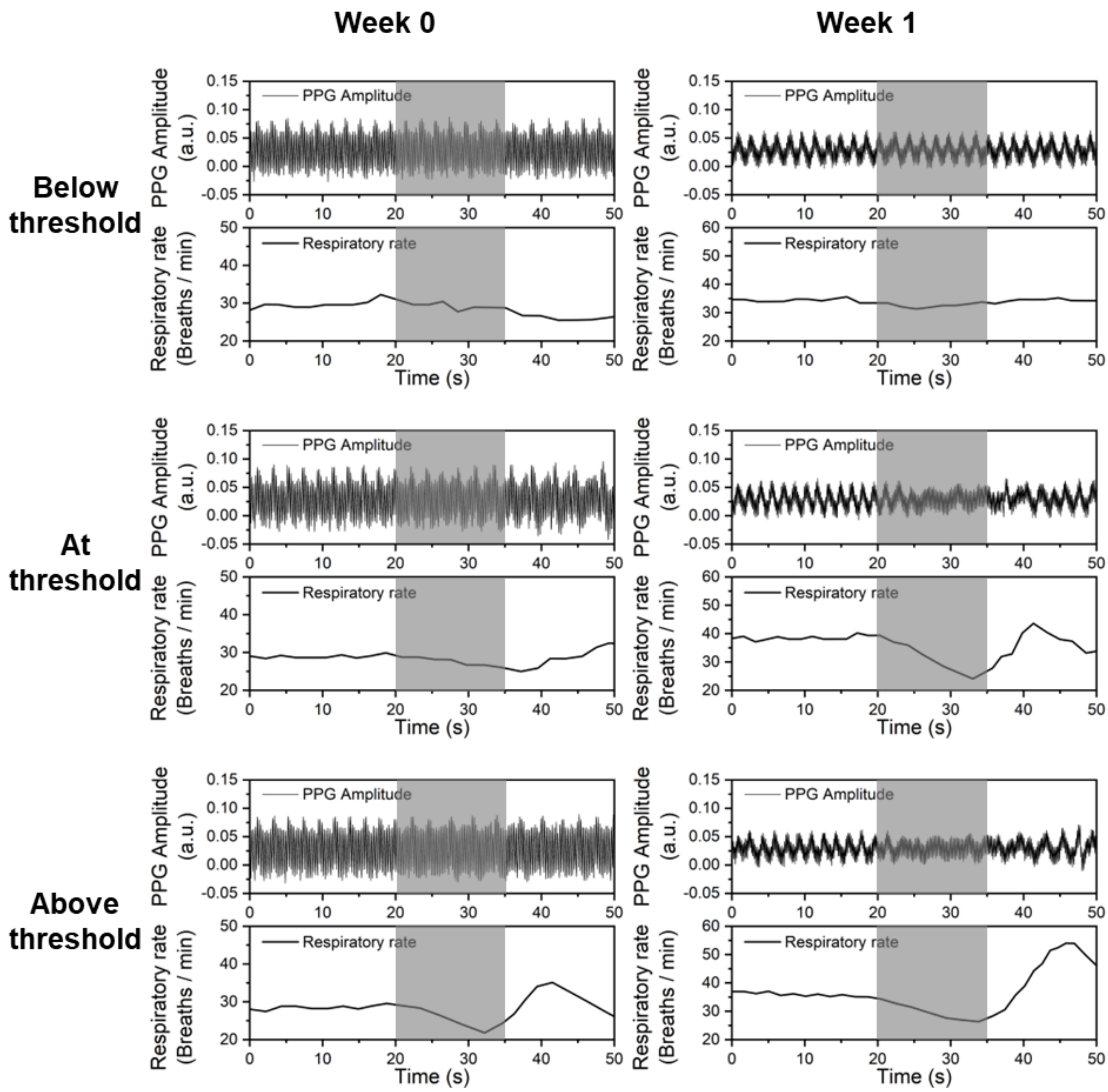

Figure S28. PPG-derived respiratory waveform recorded below (upper), at (middle), and above (lower) the functional stimulation threshold at 0 weeks and 2 weeks post-implantation. The gray region indicates the period during which vagus nerve stimulation (VNS) was applied.

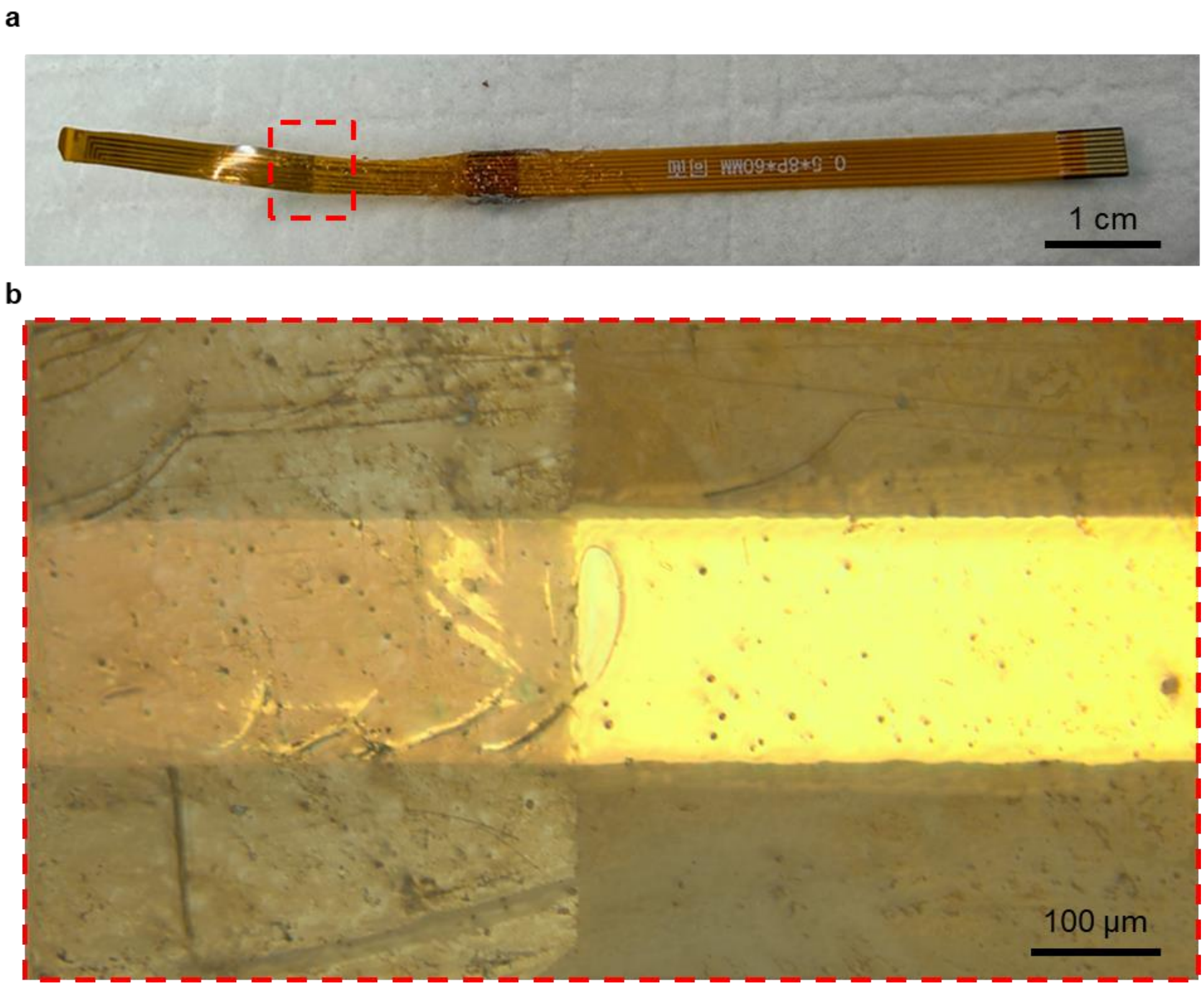

Figure S29. (a) Representative digital image of the explanted *den-2Dpt*$_{waf}$/Au electrodes after implantation. (b) Enlarged image of the area marked by the red box in (a), highlighting contrast between transferred and non-transferred regions of the *den-2DPt*$_{waf}$ film on the Au interconnect lines.

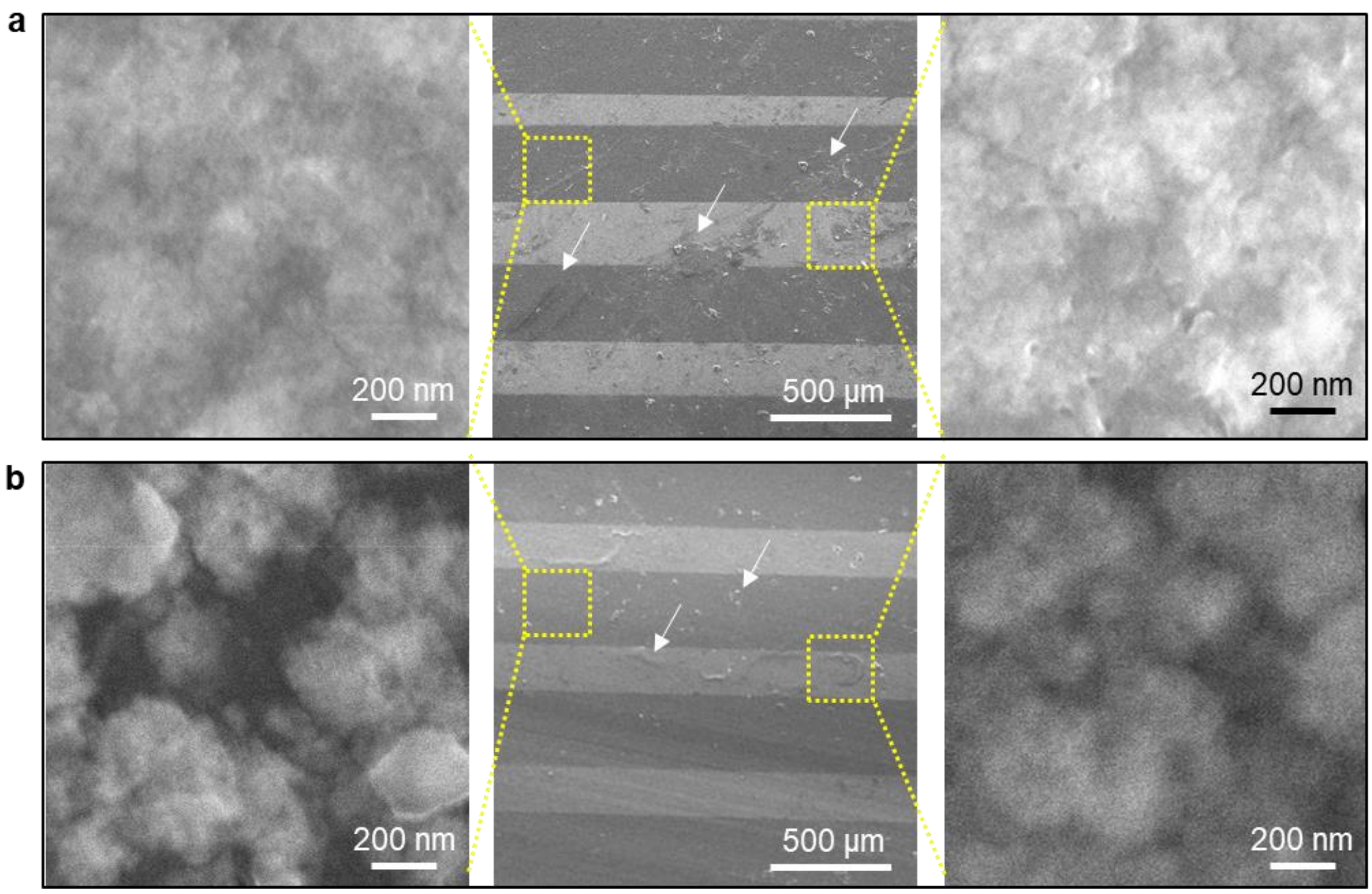

Figure S30. SEM images of the explanted *den-2DPt*$_{waf}$/Au electrode (a) as retrieved and (b) after ethanol washing with vortexing (2 min). Residual biological tissue and adherent cells (white arrows) remain on the electrode surface, partially obscuring the *den-2DPt*$_{waf}$/Au architecture and limiting direct quantitative assessment of its nanoscale morphology. Nevertheless, the overall Pt sheet structure remains continuous with no apparent macroscopic structural degradation, although quantitative evaluation is limited by the residual biological deposits.

Table S1. Comparison of the performance of *den-2DPt*$_{waf}$/Au with other representative Pt coating materials as reported in the literature.

Materials[Ref]	Electrode size/ geometry	Impedance at 1 kHz		Charge injection capacity (mC cm^{-2})	Charge storage capacity (mC cm^{-2})	Comments
		Ω	Ω cm^{2}			
***den-2DPt$_{waf}$*/Au**[This work]	Square, 200 × 200 µm^{2}; area = 4 × 10^{-4} cm^{2}	45.4	0.018	1.13	11.97	Wet-chemical scalable synthesis of intrinsically porous Pt film with clean transfer for soft and biocompatible interface; low impedance, high CSC and CIC.
Pt-black coated Pt[S3]	Pt pad, area = 5 × 10^{-3} cm^{2}	~1000	-	0.06	4.9	Sputtering based Pt-black deposition; low CIC.
nanoPt coatings[S4]	Circular disk, diameter = 35 µm; Estimated area = 9.6 × 10^{-6} cm^{2}	>1000	-	3.0	5.33	Electrochemical deposition; no significant reduction of impedance.
Pt-grass[S5]	Circular disk, diameter = 35 µm; Estimated area = 9.6 × 10^{-6} cm^{2}	>1000	-	0.3	NA	Electrochemical reduction process; low CIC and high impedance.
L_2-ePts[S6]	Circular microelectrode	-	0.039	3.0	NA	Electroplating in a reverse micelle solution; roughness dependent performance; no soft substrate integration
Porous-Pt[S7]	Circular disk, diameter = 1600 µm; Estimated area = 2.0 × 10^{-2} cm^{2}	-	2763	0.39	10.30	Electrochemical deposition; low CIC.

Table S2. Precursor's amounts and conditions used in the syntheses of various shape and pattern for *den-2DPt$_{waf}$* sheet.

Prepared Pt Sheet	Size	Substrate	Amount of Na_2PtCl_4	Amount of ascorbic acid	Amount of PVP	Reaction Conditions
den-2DPt$_{waf}$ (Circular wafer)	2-inch diameter	f-LDH/*Si* [Prepared from SL-LDH (0.2 mL, 20 mg mL)]	40.5 mL, 1.25 mM	20.3 mL, 6.7 mM	20.3 mL; 45.0 mg mL^{-1}	70 ˚C, 30 min
	3-inch diameter	f-LDH/*Si* [Prepared from SL-LDH (0.5 mL, 20 mg mL)]	91.2 mL, 1.25 mM	45.6 mL, 6.7 mM	45.6 mL, 65.0 mg mL^{-1}	

References

[S1] S. W. Jang, S. Dutta, A. Kumar, Y. R. Hong, H. Kang, S. Lee, S. Ryu, W. Choi, I. S. Lee, *ACS Nano* **2020**, *14*, 10578.

[S2] F. Wu, L. Zhang, J. Lai, W. Niu, R. Luque, G. Xu, *J. Mater. Chem. A*, **2019**, *7*, 8568.

[S3] A. R. Harris, M. Ruslim, H. Xin, Z. Shen, J. Liu, T. Spencer, D. Garrett, D. B. Grayden, S. E. John, *Sci. Rep.* **2025**, *15*, 9676–9689

[S4] C. Boehler, D. M. Vieira, U. Egert, M. Asplund, *ACS Appl. Mater. Interfaces* **2020**, *12*, 14855–14865.

[S5] C. Boehler, T. Stieglitz, M. Asplund, *Biomaterials* **2015**, *67*, 346–353.

[S6] S. Park, Y. J. Song, H. Boo, T. D. Chung, *J. Phys. Chem. C* **2010**, *114*, 8721–8726.

[S7] F. Nasirzadehroshenin, Z. Feng, *J. Electrochem. Soc.* **2024**, *171*, 112505.
